# Evaluation of Placental Transfer and Tissue Distribution of *cis*- and *Trans*-Permethrin in Pregnant Rats and Fetuses Using a Physiological-Based Pharmacokinetic Model

**DOI:** 10.3389/fped.2021.730383

**Published:** 2021-09-23

**Authors:** Stéphane Personne, Céline Brochot, Paulo Marcelo, Aurélie Corona, Sophie Desmots, Franck Robidel, Anthony Lecomte, Véronique Bach, Florence Zeman

**Affiliations:** ^1^Péritox, UMR_I 01, Université de Picardie Jules Verne, Amiens, France; ^2^Institut National de l'Environnement Industriel et des Risques (INERIS), Unité Toxicologie Expérimentale et Modélisation (TEAM), Parc ALATA BP2, Verneuil en Halatte, France; ^3^Plateforme ICAP, ICP FR CNRS 3085, Université de Picardie Jules Verne, Amiens, France

**Keywords:** pesticides, PBPK model, permethrin, pyrethroids, rat, pregnancy, fetus, brain

## Abstract

Biomonitoring studies have highlighted the exposure of pregnant women to pyrethroids based on the measurement of their metabolites in urine. Pyrethroids can cross the placental barrier and be distributed in the fetus as some pyrethroids were also measured in the meconium of newborns. Prenatal exposure to pyrethroids is suspected to alter the neurodevelopment of children, and animal studies have shown that early life exposure to permethrin, one of the most commonly used pyrethroid in household applications, can alter the brain development. This study aimed to characterize the fetal permethrin exposure throughout gestation in rats. We developed a pregnancy physiologically based pharmacokinetic (pPBPK) model that describes the maternal and fetal kinetics of the *cis*- and *trans*- isomers of permethrin during the whole gestation period. Pregnant Sprague–Dawley rats were exposed daily to permethrin (50 mg/kg) by oral route from the start of gestation to day 20. Permethrin isomers were quantified in the feces, kidney, mammary gland, fat, and placenta in dams and in both maternal and fetal blood, brain, and liver. *Cis*- and *trans*-permethrin were quantified in fetal blood and tissues, with higher concentrations for the *cis*-isomer. The pPBPK model was fitted to the toxicokinetic maternal and fetal data in a Bayesian framework. Several parameters were adjusted, such as hepatic clearances, partition coefficients, and intestinal absorption. Our work allowed to estimate the prenatal exposure to permethrin in rats, especially in the fetal brain, and to quantitatively estimate the placental transfer. These transfers could be extrapolated to humans and be incorporated in a human pPBPK model to estimate the fetal exposure to permethrin from biomonitoring data.

## Introduction

Pyrethroids are the most commonly used group of insecticides with a wide range of applications in agricultural, commercial, industrial, and residential settings. They are also used in veterinary and human medicine and for public health vector control (1). Given their broad spectrum of applications and the restriction of other classes of insecticides, their use has increased over the years, exposing a large population worldwide (2, 3). In humans, pyrethroids are rapidly metabolized in the gastro-intestinal tract and the liver and excreted as metabolites in urine (4). Permethrin metabolism involved hydrolysis by carboxylesterases and oxidation by cytochrome P450 enzymes. The exposure of human populations to pyrethroids is often based on the measurement of five urinary biomarkers: *cis*- and *trans*-3-(2,2-dichlorovinyl)-2,2-dimethyl-(1-cyclopropane) carboxylic acid, 4-fluoro-3-phenoxybenzoic acid, 3-(2,2-dibromovinyl)-2,2-dimethyl cyclopropane carboxylic acid, and 3-phenoxybenzoic acid (3-PBA). Biomonitoring studies in pregnant women have shown a widespread exposure to pyrethroids in many countries (5–11), which increased with the use of domestic insecticides (12). Pyrethroids can cross the placenta and have been measured in cord blood at delivery (7, 13, 14) and in the meconium (15–17).

According to the Developmental Origin of Health and Disease hypothesis, exposure to environmental toxicants during fetal development and childhood can contribute to the development of chronic diseases, including neurodevelopmental disorders in later stages of life (18). The nervous system is particularly vulnerable during the critical window of prenatal development due to high cellular plasticity and the differentiation of neurons or glial cells at this stage (19). Fetal exposure to pyrethroids during this critical period of brain development is of concern and could impact child neurodevelopment (20). Associations between prenatal exposure to pyrethroids and child neurobehavioral disorders have been investigated in limited studies (21–28). A positive association between pyrethroid pesticides and autism spectrum disorder, attention deficit hyperactivity disorder, or neurocognitive development has only been observed in a few studies (21, 26, 27). However, human exposure was based on the assessment of urinary metabolites, which can be common to several pyrethroids (29) and may not reflect the internal effective dose of the fetus in target tissues during critical time windows.

Among pyrethroids, permethrin [3-phenoxybenzyl (*1RS, 3RS*;*1RS, 3SR*)-*cis, trans*-3-(2,2-dichlorovinyl)-2,2-dimethylcyclopropanecarboxylate] is one of the most frequently used pyrethroids (30, 31). Permethrin is composed of a mixture of *cis*- and *trans*-isomers. Experimental studies in rodents have shown that prenatal exposure to permethrin can alter neurodevelopment and cognitive abilities (32–36). To better understand the exposure dose–response relationship, it is critical to determine the concentration of the active compound in the brain, the target tissue. Physiologically based pharmacokinetic (PBPK) models can be used to simulate internal dosimetry from an external dose and can support the extrapolations between species based on the physiological and biochemical differences (37). PBPK models for permethrin have been developed in rats (38–40) and humans at different life stages (30, 38, 40–43) but did not cover prenatal life.

In this paper, we present the development of a pregnancy pPBPK model in rats to predict the kinetic of permethrin isomers in fetal tissues and their capacity to reach the developing brain. The proposed model is an extension of our previous PBPK model for permethrin in adult rats (38). An experimental toxicokinetic study was performed in pregnant rats after single and repeated dose administrations until the end of gestation. The measured concentrations in blood and several organs and tissues were used to calibrate the gestation PBPK model in a Bayesian framework.

## Materials and Methods

### Toxicokinetic Studies in Rats

#### Chemicals

*Cis*-permethrin and *trans*-permethrin were obtained from Dr. Ehrenstorfer (Augsburg, Germany). The internal standards, ^13^C_6_-*cis*-permethrin and ^13^C_6−_*trans*-permethrin, were purchased from Cambridge Isotope Laboratories (Andover, MA, USA).

For the toxicokinetic studies, permethrin in powder form (99% purity, 40 and 60% of *cis* and *trans*-isomers, respectively) was also purchased from Dr. Ehrenstorfer. Corn oil was acquired from Sigma-Aldrich (St. Quentin Fallavier, France).

#### Animals and Experimental Design

Our experimental protocol was approved by a regional ethics committee on experiments using animals (CREMEAP no. 96) and the French Ministry of Research with the permit number 01812.01. Sprague–Dawley female rats were housed with adult males overnight (Janvier Labs, Le Genest Saint Isle, France) after a minimum of 5 days of acclimatization. Mating was confirmed by a microscopic analysis of vaginal smears on the following morning. The day when a positive vaginal smear was observed was considered as day 0 of gestation. The female rats weighed 277 ± 27 g [mean body weight (BW) ± standard deviation (SD)] at day 0. Each pregnant rat was housed in a cage with a 12-h light/12-h dark cycle. Temperature and relative humidity were maintained at 22 ± 2°C and 55 ± 15%, respectively. The animals were provided with food (Altromin rodent diet for growing animals, Genestril, Royaucourt, France) and tap water *ad libitum*. The pregnant rats were dosed orally daily by gavage with 50 mg/kg permethrin dissolved in corn oil (2 ml/kg) from the first day of gestation until the day of sacrifice at gestational day (GD) 1, GD15, or GD20. GD15 and GD20 were selected to characterize the kinetic profile in rats during the last week of pregnancy as the placental and conceptus weights increase exponentially during this period. Groups of four animals were sacrificed at 1, 2, 3, 4, 6, 10, and 24 h post-dose by an overdose intra-peritoneal injection of pentobarbital at each gestational day. The dose of 50 mg/kg (40:60 cis/trans), corresponding to 20 and 30 mg/kg of the *cis*- and *trans*-isomer, respectively, was similar to the 25 mg/kg dose used by Willemin et al. (38) in the same strain of rat. The mean body weights at GD15 and GD20 were 356 ± 24 and 422 ± 28 g, respectively, and the mean number of fetuses per litter was 13.5 ± 2.

#### Sample Collection and Chemical Analyses

At each time point, blood, whole brain, kidneys, liver, mammary glands, and abdominal fat were collected. For blood, formic acid (1%) was added v/v to blood to inhibit the metabolism of permethrin by carboxylesterases. At GD1 and GD15, the pregnant rats were kept in individual metabolic cages for 24 h to collect the feces. The placentas were collected at GD15 and GD20. Fetal blood, liver, and brain were collected at GD20. The placenta and fetal samples were pooled by litter to provide an adequate sample size for analysis. All samples were stored at −80°C until analysis.

Extraction and analyses were performed by liquid chromatography–tandem mass spectrometry (LC-MS/MS) according to the analytical method developed by our team (44). Briefly, samples of 500 mg were used except for mammary gland, fat, and feces, where a sample of 50 mg was used. For blood, an aliquot of 1.5 ml was used. The samples were transferred in vials and spiked with ^13^C_6_-*trans*-permethrin as surrogate standard. Extraction was performed by liquid–liquid extraction using acetone/hexane (2:8, vol/vol) with three consecutive extractions. The combined organic phases were evaporated to dryness under nitrogen before reconstitution for LC-MS/MS analysis. For fat, mammary gland, and feces, an additional purification step was performed using a blend of Sepra ZT-WAX and Na_2_SO_4_. All dried extracts were then reconstituted in 500 μL of acetonitrile and transferred to autosampler vials with the addition of ^13^C_6_-*cis*-permethrin as the internal standard. The analysis was conducted with an Acquity UPLC® H-Class (Waters) coupled to a triple quadrupole mass spectrometer Xevo TQ-S (Waters) with a HSS T3 column (1.8 μm; 100 | 2.1 mm, Waters). The limits of quantification (LOQs) for *cis*-permethrin were 4 ng/g in placenta and feces, 20 ng/g in the liver, brain, fat, and mammary gland, 40 ng/g in the kidneys, and 26 ng/ml in blood. The LOQs for *trans*-permethrin were 20 ng/g in the brain and placenta, 40 ng/g in feces, 80 ng/g in the liver, kidney, fat, and mammary gland, and 52 ng/ml in blood.

### Model Development

#### Model Structure

**Figure 1:**
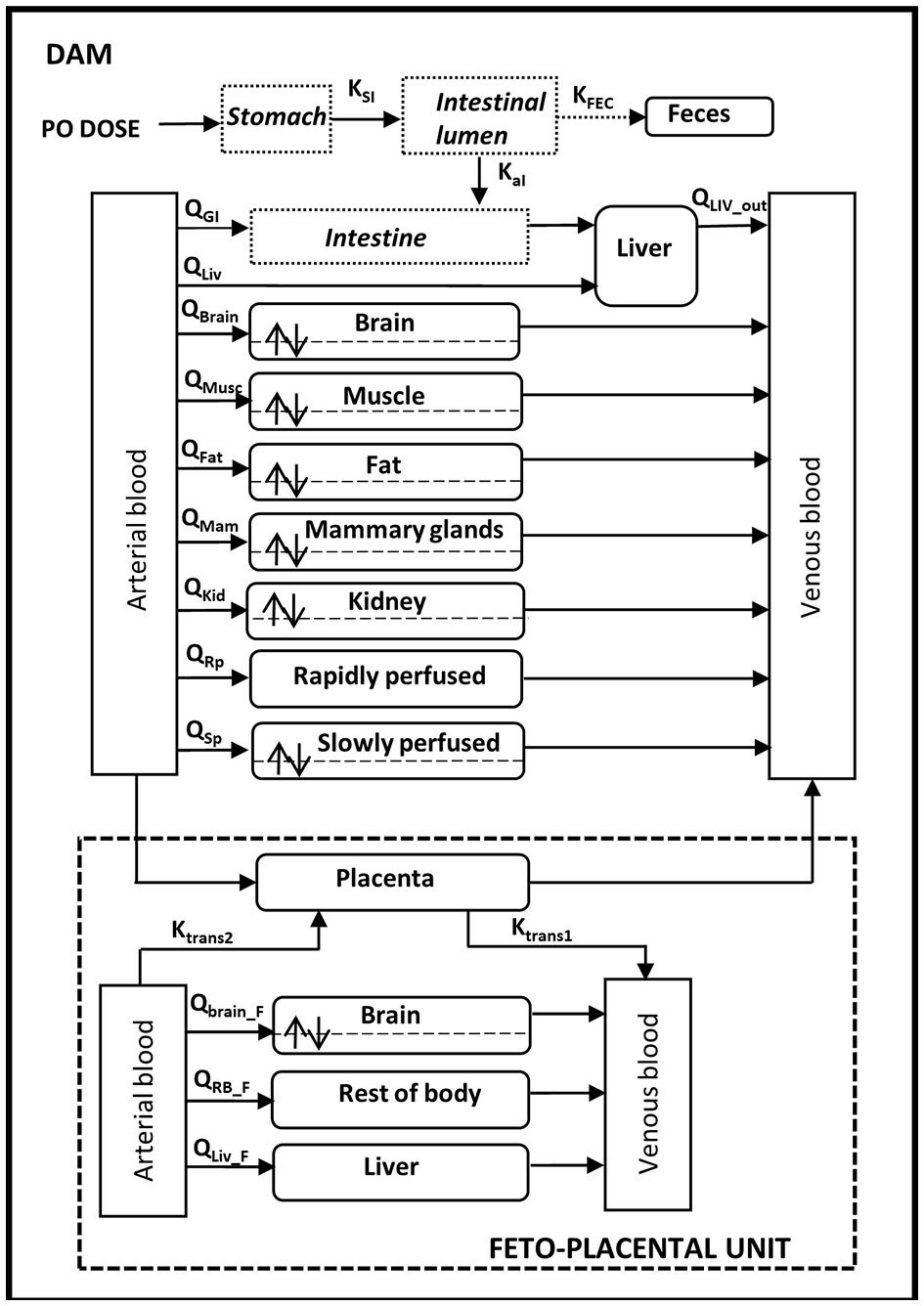
Physiologically based pharmacokinetic model of *cis*- and *trans*-permethrin in pregnant rats and fetuses.

The adult male rat PBPK model for permethrin, as published by Willemin et al. (38), was extended to include gestation. The gestational PBPK model includes a maternal sub-model and a fetal sub-model that are linked via the placenta (Figure 1). The maternal sub-model includes the same 10 compartments as the adult male model by Willemin et al. (38) (blood, brain, liver, muscle, kidney, fat, stomach, intestinal lumen, and slowly perfused and rapidly perfused tissues) except that the testes compartment was removed and the mammary gland was added as a compartment. All the model equations can be found in the former paper. The fetal sub-model includes four compartments: blood, liver, brain, and a lumped compartment for the rest of the body. All fetuses from a single litter were modeled as one large fetal model.

In pregnant rats, oral absorption was modeled using a two-compartment model with the stomach and gastro-intestinal tract and a single constant of absorption (*K*_aI_) located in the GI tract. Distribution in compartments was assumed to be limited by diffusion in the brain, muscle, fat, kidney, slowly perfused tissues, and mammary tissues because of the lipophilicity of permethrin and of the experimental observations in animals. For the intestines, liver, rapidly perfused tissue, and placenta, the distribution was blood flow-limited. Similar to the adult model, permethrin is metabolized in blood and liver and excreted in feces. Fetal exposure was through the placenta with transfers described as bi-directional between the placenta and the fetal blood. Placental transfer was modeled as a diffusion process with a first-order rate constant (*Ktrans1* and *Ktrans2*), and the amount of *cis*- or *trans*-permethrin in the placenta (*A*_Pla_) was given by:


$$\begin{array}{l} \frac{d(A_{Pla})}{dt}=Q_{Pla}\times \left(C_{Art}-\frac{C_{Pla}}{PC_{Pla}}\right)-Ktrans1\times \frac{C_{Pla}}{PC_{Pla}}\\ \;+Ktrans2\times C_{Art,F} \end{array}$$

where *Q*_Pla_ is the blood flow to the placenta, *C*_Art_ and *C*_Pla_ are the maternal arterial and placental concentrations, *PC*_Pla_ is the placenta/blood partition coefficient, and *C*_Art, F_ is the arterial concentration in the fetus. The distribution in fetal compartments was assumed to be flow-limited, with the exception of the brain. Fetal metabolism was assumed to be negligible. The model structure is identical for both isomers. The model code is provided in the Supplementary Material.

#### Model Parameterization

##### Physiological Parameters

The physiological parameters (cardiac output, blood flow, and tissue volume) for the pregnant dam and the fetus are summarized in Table 1 and were obtained from the literature or measured in this study. The model accounted for the gestation-induced changes in maternal tissue volumes and fetal growth during the whole gestation period. The equations used for the growth of maternal and fetal tissues and also the changes in blood flows occurring during gestation can be found in Supplementary Table S1.

**Table 1:** Physiological parameters of the maternal and fetal physiologically based pharmacokinetic models for *cis*- and *trans*-permethrin.

**Parameters**	**Value**	**Source**
**Pregnant rats**		
Body weight (BW, kg)	0.277–0.422^a^	This study
Cardiac output index (QCI, L/h/kg)	24.56–21.6^a^	Dowell and Kauer (45)
*Tissue volumes (fraction of initial BW, unitless)*		
Blood (*V*_Blood_)	0.06	Brown et al. (46)
GI tract (*V*_GI_)	0.027	Brown et al. (46)
Liver (*V*_Liv_)	0.0351	This study
Muscle (*V*_Musc_)	0.404	Brown et al. (46)
Brain (*V*_Brain_)	0.0073	Brown et al. (46)
Kidney (*V*_Kid_)	0.0076	Brown et al. (46)
Non-perfused (*V*_NP_)	0.05	Brown et al. (46)
Rapidly perfused (*V*_RP_)	0.046	Brown et al. (46)
Slowly perfused (*V*_SP_)	1—all organs, non-perfused	
*Tissue volume (changing during gestation, L)*		
Mammary gland (*V*_Mam_)	0.0024–0.013^a^	Hanwell and Linzell (47); Rosso et al. (48)
Fat (*V*_Fat_)	0.017–0.024^a^	Brown et al. (46); Naismith et al. (49)
Placenta (*V*_Pla_)	0–0.167^a^	Clewell (50); O'Flaherty et al. (51); Yoon et al. (52)
*Blood flows (fraction of initial cardiac output, unitless)*		
Liver (total) (*Q*_Livout_)	0.174	Brown et al. (46)
Portal (GI tract) (*Q*_GI_)	0.151	Brown et al. (46)
Arterial (GI tract) (*Q*_Liv_)	0.024	Brown et al. (46)
Muscle (*Q*_Musc_)	0.278	Brown et al. (46)
Brain (*Q*_Brain_)	0.02	Brown et al. (46)
Kidney (*Q*_Kid_)	0.141	Brown et al. (46)
Slowly perfused (*Q*_Rp_)	0.063	Brown et al. (46)
*Blood flow (changing during gestation, L/h)*		
Mammary gland (*Q*_Mam_)	0.012–0.064^a^	Hanwell and Linzell (47)
Fat (*Q*_Fat_)	0.21–0.29^a^	Clewell (50); O'Flaherty et al. (51); Yoon et al. (52)
Placenta (*Q*_Pla_)	0–1.42^a^	Clewell (50); O'Flaherty et al. (51); Yoon et al. (52)
Rapidly perfused (*Q*_Rp_)	Difference between cardiac output and the sum of theother tissue blood flows	
*Blood volume (fraction of tissue, unitless)*		
Brain (BV_Brain_)	0.03	Brown et al. (46)
Muscle (BV_Musc_)	0.04	Tornero-Velez et al. (40)
Kidney (BV_Kid_)	0.16	Brown et al. (46)
Fat (BV_Fat_)	0.02	Tornero-Velez et al. (40)
Mammary glands (BV_Mam_)	0.02	Assimilated to fat
**Fetuses**		
Body weight for individual fetus (*V*_1Fet_, kg)	0–0.0068^a^	Sikov and Thomas (53)
Cardiac output index (QCI_1F_, L/h/kg)	22.8	Girard et al. (54); Yoon et al. (52)
*Tissue volumes (fraction of BW)*		
Blood (*V*_Blood_1F_)	0.0676	Brown et al. (46)
*Tissue volume (changing during fetal growth, L)*		
Brain (*V*_Brain_1F_)	0–0.0034	Sikov and Thomas (53)
Liver (*V*_Liv_1F_)	0–0.0044	Sikov and Thomas (53)
Rest of the body (*V*_RB_1F_)	BW—sum of other tissue volumes	
*Tissue blood flow (fraction of cardiac output)*		
Brain (*Q*_Brain_1F_)	0.1055	Carter and Gu (55); Yoon et al. (52)
Liver (*Q*_Liv_1F_)	0.061	Itskovitz et al. (56); Yoon et al. (52)
Rest of the body (*Q*_RB_1F_)	Cardiac output—sum of other tissue blood flows	
*Blood volume (fraction of tissue)*		
Brain (BV_Brain_1F_)	0.03	Set to adult value from Willemin et al. (38)
Rest of the body (BV_RB_1F_)	0.05	Set to adult value from Willemin et al. (38)

a *Range during the gestation period*.

##### Chemical Specific Parameters

Chemical specific parameters for pregnant dams and fetuses were estimated using the measured concentrations in our toxicokinetic study, with the exception of the rate constants for absorption and for blood and intestinal metabolism. The parameters were estimated simultaneously in a Bayesian calibration framework using our experimental data. Separate calibrations were performed with data generated at GD1 or at GD15/GD20 after repeated daily administration. In a Bayesian approach, all parameters are considered as random variables. A prior distribution was defined for each parameter based on the knowledge on the values of the parameter. In conjunction with a likelihood function of the data, a posterior distribution was determined by random sampling methods (57).

For absorption, two metabolic rate constants are considered in the model: the intestinal absorption rate constant (*K*_aI_) and the stomach–intestine transfer rate constant (*K*_sI_). As it was not possible to estimate both parameters based on our experimental data, the *K*_aI_ values of the adult model of Willemin et al. (38) were used. Only the *K*_sI_ values were estimated as *K*_sI_ is considered as a sensitive parameter according to previous PBPK models published for pyrethroids (58). For metabolic clearances, as the liver is the main site of metabolism (59), only metabolic rate constants for permethrin isomers in the liver were estimated. Blood and intestinal rate constant values were set to the optimized values of the adult model of Willemin et al. (38).

A truncated normal distribution was assigned to the parameters for which values were reported in previous PBPK models for permethrin in adult rats (38, 40). The mean values of the prior distribution were taken from the PBPK model of Willemin et al. (38), with the exception of the liver clearance for which the values of the PBPK model of Tornero et al. (40) were used. For all other parameters, a uniform distribution was applied. The coefficients of variation were set to 50% for all parameters. The prior distributions of all parameters are reported in Table 2. The likelihood of the data was assumed to follow a lognormal distribution with 15% of error. The posterior distributions were estimated by Markov Chain Monte Carlo simulations using MCSim (ver.5.6.6) software. Three independent Markov chains of 10,000 iterations were run, and one in two of the last 4,000 iterations were recorded to check the convergence using the potential scale reduction factor *R^*. An acceptable convergence was considered as reached when the *R^* value was 1.2 or less (60).

**Table 2:** Distribution of the chemical specific parameters of the model for permethrin isomers in pregnant rats and fetuses.

**Parameters**	**Prior distribution**		**Posterior distribution**		
	***cis*-Permethrin**	***trans*-Permethrin**		***cis*-Permethrin**	***trans*-Permethrin**
**Pregnant dam**					
*Partition coefficients*					
Liver: blood (PC_Liv_)	0.89 ± 0.445 (10^−3^-20)	(10^−3^-20)	GD1	**2.33** (2.14–2.61)	–
			GD15/20	**1.48** (1.37–1.61)	**1.36** (1.19–1.61)
GI: blood (PC_GI_)	Equal to PC_Kid_	Equal to PC_Kid_	–	–	–
Fat: blood (PC_Fat_)	225 ± 112.5 (5–900)	76 ± 38 (5–900)	GD1	**345** (195–592)	**60.5** (28.9–154)
			GD15/20	**545** (414–744)	**165** (119–234)
Mammary gland: blood (PC_Mam_)	225 ± 112.5 (5–900)	76 ± 38 (5–900)	GD1	**212** (65.8–487)	**5.09** (5–5.52)
			GD15/20	**436** (312–654)	**46.7** (42.9–52.1)
Muscle: blood (PC_Musc_)	Fixed to 1.2	Fixed to 0.82	–	–	–
Brain: blood (PC_Brain_)	1.60 ± 0.80 (10^−3^-20)	0.57 ± 0.285 (10^−3^-20)	GD1	**2.67** (2.37–3.12)	**0.72** (0.39–1.34)
			GD15/20	**1.15** (1.08–1.26)	**0.64** (0.58–0.73)
Kidney: blood (PC_Kid_)	1.10 ± 0.55 (10^−3^-20)	Fixed to 0.21	GD1	**5.61** (5.09–6.30)	–
			GD15/20	**3.00** (2.78–3.32)	–
Placenta: blood (PC_Pla_)	(10^−3^-20)	(10^−3^-20)	GD15/20	**3.52** (3.14–4.03)	**3.00** (2.67–3.47)
Slowly perfused: blood (PC_Sp_)	19 ± 9.50 (10^−3^-20)	8.4 ± 4.20 (10^−3^-20)	GD1	**14.7** (5.5–20.0)	**9.5** (3.2–18.8)
			GD15/20	**4.55** (3.42–6.21)	**1.55** (0.35–12.8)
Rapidly perfused: blood (PC_Rp_)	Fixed to 1.1	Fixed to 0.21	–	–	–
*Permeability coefficients (L/h)*					
Brain (PS_Brain_)	1.0. ± 0.5.10^−3^ (10^−5^-1)	1.2. ± 0.6.10^−3^ (10^−5^-1)	GD1	**5.6.10**^**−3**^(5.2–6.3.10^−3^)	**1.6.10**^**−3**^(1.1–2.6.10^−3^)
			GD15/20	**3.5.10**^**−3**^(3.1–4.1.10^−3^)	**2.2.10**^**−3**^(1.8–2.8.10^−3^)
Kidney (PS_Kid_)	(10^−5^-1)	(10^−5^-1)	GD1	**1.3.10**^**−2**^(1–2.10^−2^)	**1.3.10**^**−2**^(1–2.10^−2^)
			GD15/20	**0.24** (0.14–0.3)	**0.24** (0.14–0.3)
Fat (PS_Fat_)	4.8 ± 2.4.10^−2^ (10^−5^-1)	0.11 ± 0.055 (10^−5^-1)	GD1	**0.10** (0.09–0.11)	**0.07** (0.06–0.09)
			GD15/20	**4.5.10**^**−2**^(4–5.4.10^−2^)	**9.2.10**^**−3**^(8.3–10.6.10^−3^)
Mammary gland (PS_Mam_)	(10^−5^-1)	(10^−5^-1)	GD1	**0.252** (0.23–0.283)	**0.522** (0.443–0.631)
			GD15/20	**0.125** (0.112-0.147)	**0.253** (0.172–0.368)
Muscle (PSMusc)	Fixed to 0.32	Fixed to 0.48	–	–	–
Slowly perfused (PS_Sp_)	0.31 ± 0.055 (10^−5^-1)	0.065 ± 0.032 (10^−5^-1)	GD1	**0.20** (0.02–0.55)	**0.07** (0.02–0.15)
			GD15/20	**0.79** (0.58–0.99)	**0.11** (0.03–0.19)
*Rate constant (h^−1^)*					
Stomach–intestine transfer (*K*_si_)	0.35 ± 0.175 (0–2)	0.20 ± 0.10 (0–2)	GD1	**0.15** (0.14–0.16)	**0.058** (0.043-0.071)
			GD15/20	**0.18** (0.17–0.19)	**0.084** (0.075–0.096)
Intestinal absorption (*K*_ai_)	Fixed to 0.52	Fixed to 1.30	–	–	–
Fecal excretion (*K*_Fec_)	0.39 ± 0.195 (0–2)	0.85 ± 0.42 (0–2)	GD1	**0.07** (0.06–0.10)	**0.09** (0.07–0.12)
			GD15/20	**0.020** (0.018–0.024)	**0.033** (0.028–0.040)
*Metabolic clearance (L/h/kg)*					
Intestinal metabolism (CL_GI_)	Fixed to 0.04	Fixed to 0.3	–	–	–
Blood metabolism (CL_Blood_)	Fixed to 0.07	Fixed to 0.29	–	–	–
Liver metabolism (CL_Liv_)	6.20 ± 3.10 (1–15)	24.30 ± 12.15 (1–50)	GD1	**8.44** (7.92–9.10)	**19.4** (15.7–23.6)
			GD15/20	**2.40** (2.3–2.6)	**20.5** (19.3–22.2)
*Placental transfer (L/h/kg ^0.75^)*					
Dam to fetus (scK_trans1_)	(0–6)	(0–6)		**1.91** (1.39–2.65)	**1.91** (1.39–2.65)
Fetus to dam (scK_trans2_)	(0–6)	(0–6)		**2.52** (1.85–3.22)	**2.52** (1.85–3.22)
**Fetuses**					
*Partition coefficients*					
Liver: blood (PC_Liv_F_)	(10^−3^-20)	(10^−3^-20)		**5.41** (4.97–6.11)	**5.41** (4.97–6.11)
Brain: blood (PC_Brain_F_)	(10^−3^-20)	(10^−3^-20)		**2.01** (1.84–2.24)	**2.01** (1.84–2.24)
Rest of the body: blood (PC_RB_F_)	(10^−3^-900)	(10^−3^-900)		**57.20** (45–75.5)	**57.20** (45–75.5)
*Permeability coefficients (L/h)*					
Brain (PS_Brain_F_)	(10^−5^-1)	(10^−5^-1)		**7.9.10**^**−3**^(5.9–14.2.10^−3^)	**7.9.10**^**−3**^ (5.9–14.2.10^−3^)

*Posterior distributions are represented by mean (value in bold) with the 2.5th and 97.5th percentiles*.

#### Sensitivity Analyses

A global sensitivity analysis (GSA) using the Sobol method was conducted on the PBPK model to identify the compound-specific parameters that have the most impact on the internal maternal and fetal exposures of permethrin (*cis*-isomer) following the same exposure scenario of the *in vivo* experiments. Three model outputs were selected: the arterial concentrations of the mother and the fetus and the concentration in the fetal brain. Truncated normal distributions were assigned to the specific compound parameters, with a mean estimated mean value (Table 2) and a coefficient of variation of 30%. The SA was run at three time points at GD15 and GD20 (4, 6, and 12 h after the oral administration). The SA results are presented as two indices: the first order index, which is the variance contribution of one parameter to the total model variance, and the total order index, which is the result of the main effect of the parameter and of its interactions with the other parameters.

## Results

### Toxicokinetic Profiles in Pregnant Rats and Fetuses

**Figure 2:**
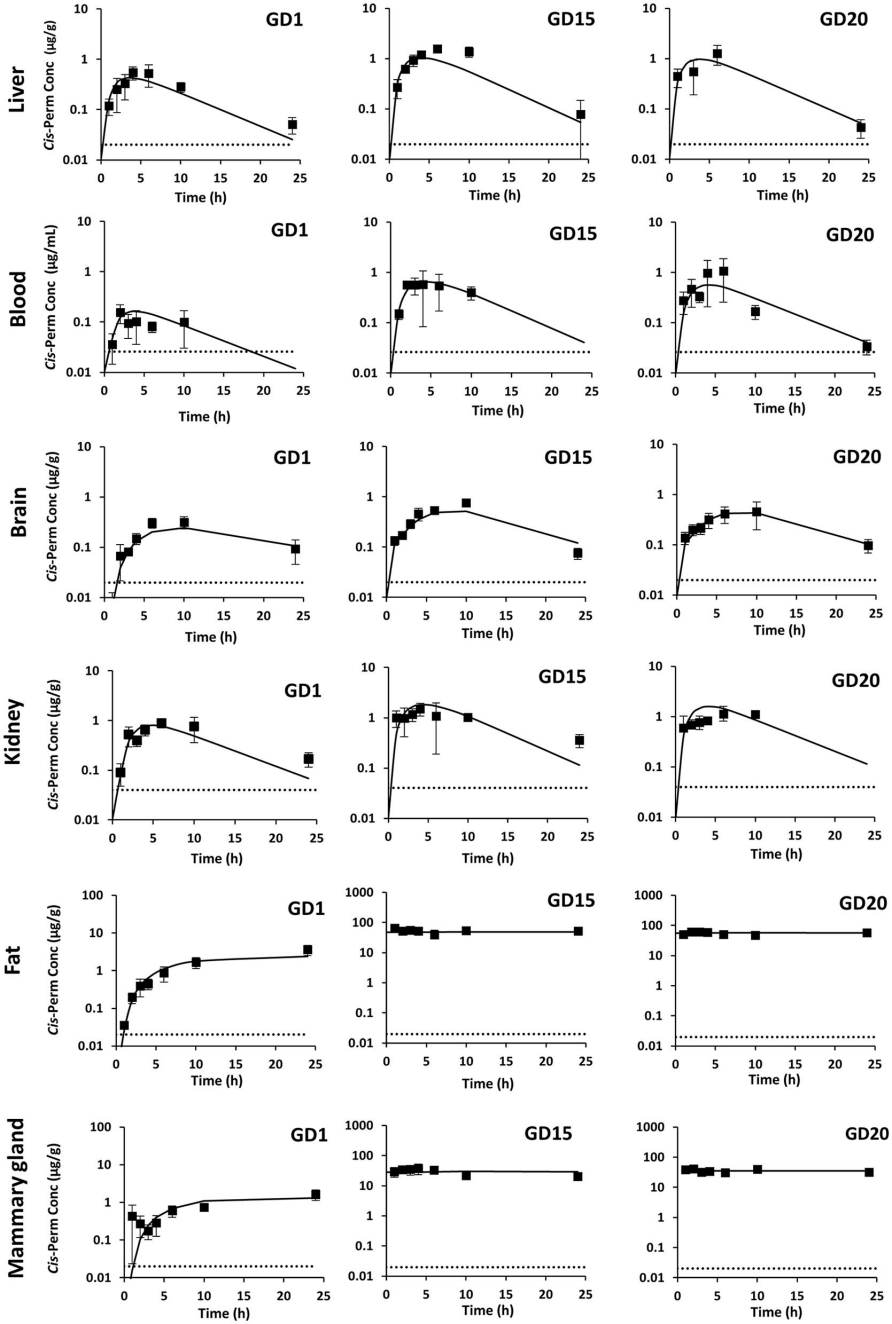
Measured concentrations (squares) and toxicokinetic profiles estimated with the physiologically based pharmacokinetic model (solid line) of *cis*-permethrin in pregnant rats at GD1, GD15, and GD20. The gray dotted line stands for the limit of quantification.

**Figure 3:**
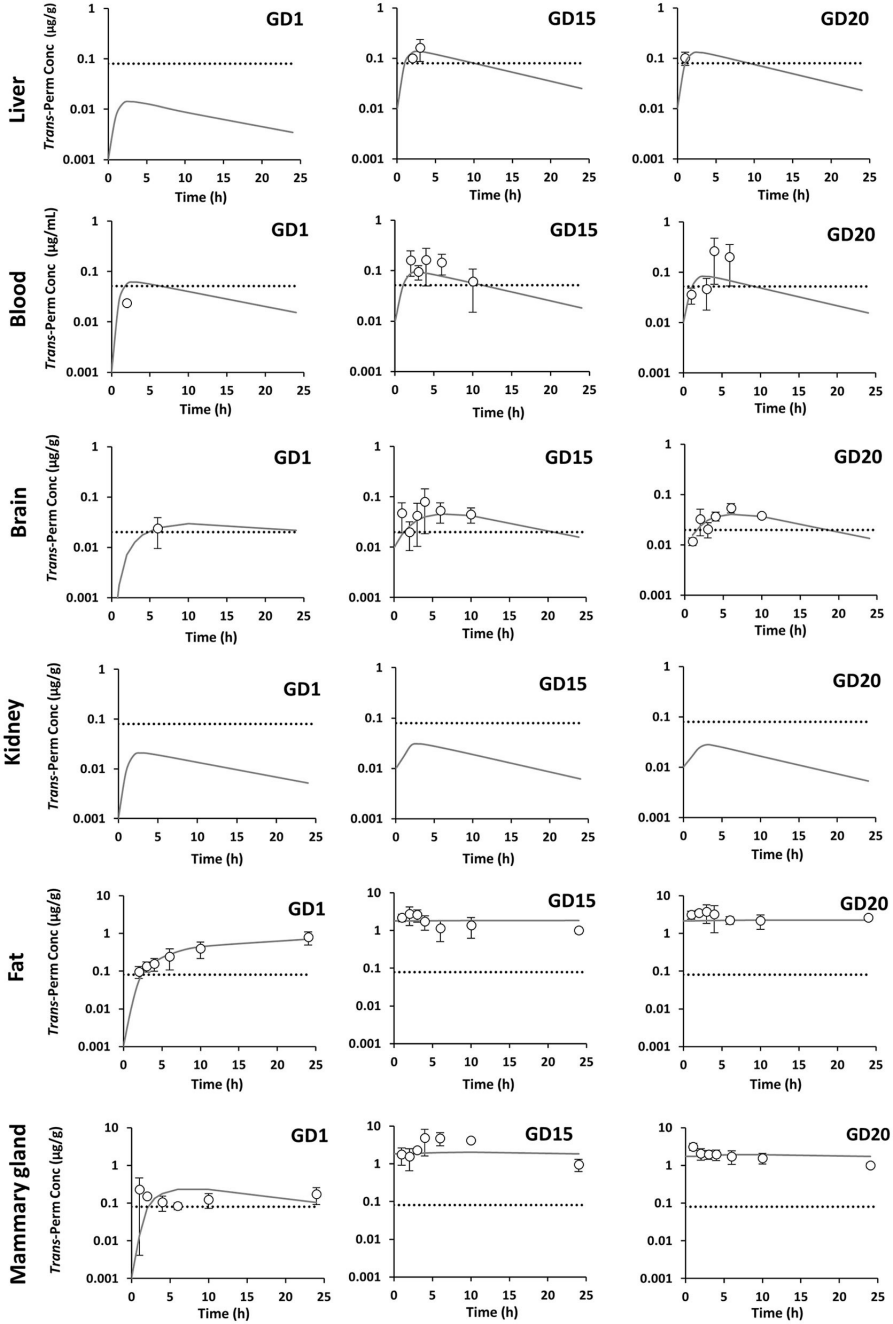
Measured concentrations (dots) and toxicokinetic profiles estimated with the physiologically based pharmacokinetic model (solid line) of *trans*-permethrin in pregnant rats at GD1, GD15, and GD20. The gray dotted line stands for the limit of quantification.

**Figure 4:**
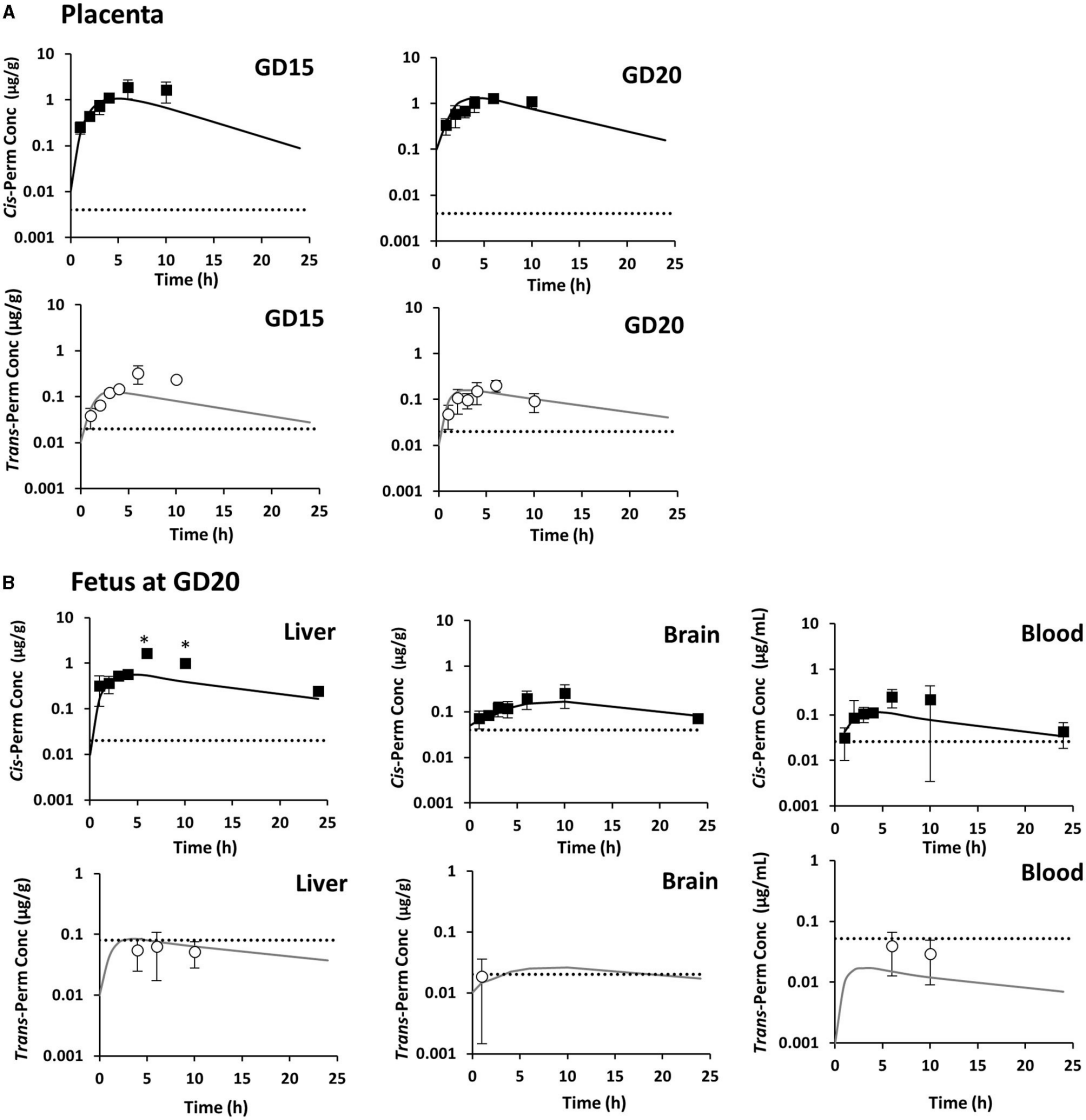
Measured concentrations of *cis-*permethrin (squares) and *trans-*permethrin (dots) and toxicokinetic profiles estimated with the physiologically based pharmacokinetic model (solid lines) in the placenta **(A)** and fetal tissues **(B)**. Mean values ± SD for *n* = 4 at each time point except for the point marked with an asterisk (*n* = 1). The gray dotted line stands for the limit of quantification.

In pregnant rats, *cis*-permethrin was quantified in blood, feces, and all tissues after a single administration at GD1 and repeated administrations until GD15 and GD20. Because *trans*-permethrin is eliminated more rapidly from the body than *cis*-permethrin, *trans*-permethrin was not quantified in several samples. Quantification was performed at the three time points studied (GD1, GD15, and GD20) in fat, mammary gland, and feces and only performed at GD15 and GD20 in blood, brain, and liver. *Trans*-permethrin was not quantified in the kidneys. For the samples where compounds were detected but below the LOQ, the concentration was set to LOQ/2. The kinetic profiles of *cis*- and *trans*-permethrin in blood and tissues at GD1, GD15, and GD20 in pregnant rats are presented in Figures 2, 3, respectively. *Cis-*permethrin was quantified in placenta, fetal blood, fetal liver, and fetal brain, whereas *trans*-permethrin could only be quantified in placenta and fetal brain, even if it could be detected in fetal liver and blood (Figure 4).

For *cis*-permethrin, after a single administration at GD1, the maximal concentration (*C*_max_) was reached between 4 and 6 h in blood, liver, and kidney and declined rapidly, with an observed half-life of 3.7, 5.7, and 6.5 h (Table 3). In the brain, fat, and mammary gland, a slower diffusion was observed, with the peak concentrations occurring later; at 10 h in the brain and at 24 h in fat and mammary gland. In the brain, the estimated half-life was 7.9 h. After repeated administration, there were no significant changes in *T*_max_ values at GD15 and GD20. However, the values of half-life were reduced compared to GD1, with the lowest values reported at GD15.

**Table 3:** Maximum time concentration (*T*_max_, h)/half-lives (*T*_1/2_, h) of *cis*- and *trans*-permethrin in the blood and tissues of pregnant rats at GD1, GD15, and GD20.

	***cis*** **-Permethrin**						***trans*** **-Permethrin**					
	**GD1**		**GD15**		**GD20**		**GD1**		**GD15**		**GD20**	
	**T_**max**_**	**T_**1/2**_**	**T_**max**_**	**T_**1/2**_**	**T_**max**_**	**T_**1/2**_**	**T_**max**_**	**T_**1/2**_**	**T_**max**_**	**T_**1/2**_**	**T_**max**_**	**T_**1/2**_**
Blood	6	3.7	4	2.6	6	3.6	–	–	4	3.2	4	–^a^
Liver	4	5.7	6	3.4	6	3.7	–	–	3	–^a^	–	–
Brain	10	7.9	10	4.2	10	6.3	–	–	4	5.4	2	–^a^
Kidney	6	6.5	4	6.7	6	–^a^	–	–	–	–	–	–
Fat	24	–^a^	1	–^a^	2	–^a^	10	–^a^	3	–^a^	3	–^a^
Mammary gland	24	–^a^	4	–^a^	10	–^a^	1	–^a^	4	–^a^	1	–^a^
Placenta	na	na	6	3.2	6	4.5	na	na	6	–^a^	6	–^a^

*–a, no half-life was computed due to insufficient data in the elimination phase.*

*na, not applicable.*

*–, no compound was quantified*.

In order to compare maternal and fetal exposure, the 24-h area under the curve (AUC_obs_) for *cis*-permethrin in blood and tissues was computed using the measured concentrations in pregnant rats and fetuses and are presented in Tables 4, **6**, respectively. In pregnant dams, the fat and the mammary gland had the highest AUC_obs_, which were respectively 44- and 21-fold higher to that in blood at GD1 (Table 4). No significant accumulation was observed after repeated administration in the kidney, liver, and brain, with AUC_obs_ ratios between GD1 and GD15 or GD20 values ranging from 1.5 to 3.4. On the contrary, in blood, fat, and mammary gland, the AUC_obs_ values at GD15 and GD20 were increased by 5.3- and 6-fold in blood, 28.4- and 28.7-fold in fat, and 28.6- and 40.3-fold in mammary gland compared to GD1. The AUC_obs_ in placenta for the *cis*-isomer was similar at GD15 and GD20 and was 3.6- and 2.3-fold the AUC_obs_ in blood, respectively (**Table 6**). In fetuses, the liver had the highest exposure, and the lowest exposure was observed in blood. The exposure in fetal blood and fetal brain was lower than the exposure of the dam to *cis*-permethrin. In the brain, the AUC ratio (fetus/dam) was 0.54. On the contrary, the exposure in the fetal liver was 1.6 times greater than the exposure of the dam in the liver.

**Table 4:** Area under the curve (μg h/ml or μg h/g) of the observed concentrations (AUC_obs_) and the estimated concentrations (AUC_est_) for *cis*-permethrin in the blood and tissues of pregnant rats.

	**GD1**		**GD15**		**GD20**	
	**AUC_**obs**_**	**AUC_**est**_**	**AUC_**obs**_**	**AUC_**est**_**	**AUC_**obs**_**	**AUC_**est**_**
Blood	1.26	1.60	6.71	7.72	7.52	6.53
Liver	6.20	4.72	21.22	11.79	15.79	10.75
Brain	4.80	3.88	10.28	8.07	7.05	6.86
Kidney	12.89	9.45	20.34	21.59	–	18.26
Fat	43.97	37.18	1,247.08	1,171.88	1,260.18	1,357.83
Mammary gland	21.23	22.06	606.71	690.07	856.41	840.45
Placenta	na	na	23.92	12.80	17.67	16.13

*–, no data available.*

*na, not applicable*.

Regarding *trans*-permethrin, the concentrations of the *trans*-isomer were always lower than those measured for the *cis*-isomer even if the administration of the *trans* isomer was slightly higher than the *cis*-isomer. For the *trans*-isomer, due to the low number of time points for which the concentration measured was above the LOQ, AUC_obs_ could only be estimated in pregnant rats at GD15 and GD20 in blood, brain, and fat and at GD1, GD15, and GD20 in the mammary gland (Table 5). The highest exposures were observed in fat and mammary gland as identified for the *cis*-isomer and were 29- and 62-fold and 59- and 36-fold the AUC_obs_ in blood at GD15 and GD20, respectively.

**Table 5:** Area under the curve (μg h/ml or μg h/g) of the observed concentrations (AUC_obs_) and the estimated concentrations (AUC_est_) for *trans*-permethrin in the blood and tissues of pregnant rats.

	**GD1**		**GD15**		**GD20**	
	**AUC_**obs**_**	**AUC_**est**_**	**AUC_**obs**_**	**AUC_**est**_**	**AUC_**obs**_**	**AUC_**est**_**
Blood	–	0.42	1.18^a^	0.75	1.04^a^	0.61
Liver	–	–	–	1.54	–	0.95
Brain	–	0.52	0.77^a^	0.71	0.53^a^	0.59
Kidney	–	–	–	–	–	–
Fat	10.60^a^	10.06	34.41	44.10	61.77	53.29
Mammary gland	–	3.94	73.20	46.66	37.53	44.18
Placenta	na	na	–	1.72	–	2.26

a *Value of extrapolated AUC.*

*na, not applicable.*

*–, no data available*.

### Model Calibration

#### Convergence Analysis and Posterior Distributions

The convergence criterion *R^* was computed for the three chains for all parameters calibrated with datasets of GD1 or GD15 and GD20. All the *R^* values were below 1.2, indicating that the convergence was reached in both cases. The posterior distributions of the estimated parameters at GD1 or GD15/GD20 are reported in Table 2 as the mean with 95% confidence interval. The means of the posterior distributions of the estimated parameters at GD1 or GD15/GD20 were compared to prior distribution and between them.

At GD1, the estimated means were close to prior estimates for tissue/blood partition coefficient (PC) in slowly perfused tissue for both isomers and for the hepatic clearance of the *trans*-isomer only. For the *cis*-isomer, the hepatic clearance was modestly increased by 36%. For all other parameters, the posterior means differed from their prior values. A decrease was observed for the parameters of absorption (*K*_sI_) and fecal excretion (*K*_Fec_), with a decrease of 57 and 72% for *K*_sI_ and 82 and 90% for *K*_Fec_ for *cis*- and *trans*-permethrin, respectively. On the contrary, the PC values were markedly increased for *cis*-permethrin. The mean PC values for the posterior distribution were 1.5-, 2.6-, and 5.1-fold higher than the prior mean values for fat, liver, and kidney.

When compared together, the estimated means at GD15 and GD20 were substantially different from those calibrated with concentration data at GD1, with the exception of *K*_sI_ values for both isomers and also hepatic clearance and PC in brain for *trans*-permethrin. The mean values of all parameters were decreased compared to GD1 values, with the exception of the PC in fat and mammary gland and permeability coefficients in slowly perfused tissue. For *cis*-permethrin, the hepatic clearance and fecal excretion were decreased by 3.5- and 3.6-fold, respectively. The PCs were decreased by a factor of 1.6, 1.9, 2.3, and 3.2 in the liver, kidney, brain, and slowly perfused tissues, respectively.

In fetuses, the PCs in the brain and liver were highest than their respective values in dams. Asymmetric placental rate constants were observed between maternal placenta and fetal blood, with the majority of the transfer being in the fetal-to-maternal direction. A 0.76 ratio was estimated between transfer rates from the dam to the fetus (scKTrans1) and from the fetus to the dam (scKTrans2).

#### Comparison of Predictions With Experimental Data

The experimental data at GD1, GD15, and GD20 were compared with the model predictions with estimated parameters at GD1 or at GD15 and GD20 (Figures 2–4). The estimated concentrations were generally in good agreement with the observed data in blood and tissues in dams. The estimated and observed AUC in pregnant rats and fetuses are reported in Tables 4–6. The estimated to observed AUC ratios for *cis*- and *trans*-permethrin were within a range between 0.5 and 1.5, indicating acceptable prediction results with the exception of fetal liver. For fetal liver, the AUC ratio was 0.46. However, at time points +6h and +10h, *cis*-permethrin was only quantifiable in one sample.

**Table 6:** Area under the curve (μg h/ml or μg h/g) of the observed concentrations (AUC_obs_) and the estimated concentrations (AUC_est_) for *cis*-permethrin in fetal blood, liver, and brain.

	***cis*** **-Permethrin**	
	**AUC_**obs**_**	**AUC_**est**_**
Blood	3.35	1.66
Liver	17.86	8.18
Brain	3.83	2.94

Using the model, it was possible to estimate AUC for *trans*-permethrin in pregnant rats notably after a single administration at GD1 even if the lack of measured concentrations prevents the computation of an observed AUC_obs_ (Table 5). At GD1, the tissue/blood ratios for AUC_est_ were 1.2 in the brain and 9.4 and 24 in mammary gland and fat, respectively. In fat and mammary gland, these ratios were ~2-fold lower than those observed with *cis*-permethrin, similar to the observed ratio in fat in toxicokinetic studies performed in male rats with permethrin (*cis*/*trans*, 40:60) (40).

Based on AUC_est_, the AUC values for the *cis*-isomer were 3.7- to 5.6-fold greater than that of the *trans*-isomer in blood and tissues at GD1. At GD15 and GD20, the *cis/trans* AUC_est_ ratios were increased compared to those calculated at GD1.

### Sensitivity Analyses

The GSA identified the parameters to which the maternal and fetal blood concentrations and fetal brain concentrations are sensitive at GD15 and GD20. Three parameters mostly influenced the maternal blood concentration at the three time points (4, 6, and 12 h after the oral administration), i.e., the hepatic clearance and absorption parameters, partition coefficient (Supplementary Figure S1). The influence of the other model parameters is quite negligible. Regarding the fetal dosimetry in blood and brain, the hepatic clearance is still the most influential parameter (Supplementary Figures S2, S3). The absorption parameters also have an impact on the blood concentration, with a decreasing influence over time. As it could have been expected, the fetal blood and brain concentrations are sensitive to the two parameters driving the placental transfer (*K*_*trans*1_ and *K*_*trans*2_). Due to its high volume, the compartment “rest of the body” also influences the kinetics in fetal blood. In the brain, the partitioning in the tissues (*PC*_*BrainF*_) and the permeability (*PS*_*BrainF*_) become influential parameters. For all model outputs, the rankings of the parameters by the first order and total order indices were similar, and significant interactions were observed between the most influential parameters.

## Discussion

Gestation induces numerous physiological, biochemical, and metabolic changes that can affect the disposition of xenobiotics (61). Assessing the fetal exposure during the whole gestation will then require careful considerations of the maternal exposure. In this paper, we extended the structure of a PBPK model for permethrin in adult male rats (38, 40) to integrate the dynamic changes occurring during gestation. Published toxicokinetic data for permethrin isomers were obtained in male rats after a single-dose administration (38, 40, 62). The existence of gender differences in kinetics of pyrethroids has not been experimentally studied for the parent compounds but only for some metabolites, 3-phenoxybenzyl alcohol and 3-phenoxybenzaldehyde, in rats for which gender differences were observed (63). To characterize these potential differences for permethrin, we collected toxicokinetic data after a single oral dose at the first day of gestation (GD1), as the modifications due to gestation are supposed to be negligible at GD1.

Compared to the known toxicokinetic profile of permethrin in male rats, female rats demonstrated a similar hepatic clearance but with a slower absorption rate. Indeed the estimated stomach intestine rate transfers (*K*_si_) in females were 2.3- and 3.6-fold lower compared to those in males for *cis-* and *trans-*permethrin, respectively. These results are in accordance with the sex differences in the gastrointestinal tract that have been reported in rats (64) and the higher gastric mucosal blood flow observed in male than in female rats (65).

*Cis*- and *trans*-permethrin were mainly distributed in tissues, with a high accumulation in fat and mammary glands. The estimated AUCs for *cis*- and *trans*-permethrin in blood (normalized by the dose) were similar to those reported by Tornero et al. (40) at 10 mg/kg but 2.7 and 5.7 lower than those reported in Willemin et al. (38) at 25 mg/kg for each isomer, respectively. However, in the study of Willemin et al., the blood concentrations exceeded the binding capacity of rat plasma, impacting the estimation of partition coefficients (66). In our study, the observed *C*_max_ in blood for *cis*-permethrin was 260 nM, in the linear range of the binding in plasma. Highest tissue/blood partitions coefficients were estimated in our study compared to those reported for males in the study of Tornero et al. (40). In rats, *cis*- and *trans*-permethrin are primarily bound by plasma proteins (50–60%) and, to a lesser amount, by lipoproteins (30–35%) (67). Sex differences in plasma apolipoprotein profiles have been reported in rats, with higher concentrations observed in males than in females (68), which could explain these differences observed between the studies.

Using estimated parameters at GD1, corresponding to a non-pregnant rat, the model was not able to capture the toxicokinetic at GD15 and GD20, demonstrating an impact of gestation on the toxicokinetic profile of permethrin. The main observed differences were related to hepatic clearance and tissue/blood PC. The predicted hepatic clearance of *cis*-permethrin at GD15 and GD20 was reduced by 3.5 compared to the prediction at GD1.

The calibration was performed in a Bayesian interference framework, allowing the integration of informative prior knowledge on the parameters and experimental data to optimize the model parameter values and inform on their variability. The sensitivity analysis identified hepatic clearance, absorption parameters, and partition coefficients as having the most impact on blood and brain concentrations. These results were in agreement with previously published sensitivity analyses on the kinetics of permethrin and deltamethrin in rodents or humans (41, 42, 58). Because the model was not able to capture the kinetics observed at GD1, GD15, and GD20 with the same set of parameter values, the model was calibrated independently with the data generated at GD1 after a single-dose administration and the data generated at GD15 or GD20 after repeated daily yielding to two different sets of parameters for GD1 and GD15/GD20. The main differences between these two sets of estimated values were related to the hepatic clearance and the tissue/blood partition coefficients. The predicted hepatic clearance of *cis*-permethrin at GD15 and GD20 was reduced by a 3.5 factor compared to the value estimated at GD1.

Gestation is associated with a small decrease in total rodent liver P450 content and/or activity (69, 70), which can explain these differences in the metabolic rate of the *cis*-isomer. For *trans*-permethrin, it was not possible to observe such a reduction due to the limited number of time points at which the isomer was quantified. The respective values of the PCs for fat and mammary gland were markedly increased by 1.6 and 2.1 for *cis*-permethrin and 2.7 and 9.2 for *trans*-permethrin. This evolution might be explained by the fact that blood lipid levels can increase up to 4-fold during gestation (71) and affect the disposition of lipophilic compounds such as permethrin [log *P* = 6.1; (40)]. Moreover, maternal fat content progressively increases during gestation and mammary fat accumulation increases intensely from day 12 of gestation, contributing to maternal fat storage (48, 72). These differences in blood and tissue composition during the gestation may explain the changes in PC values and the changes in pharmacokinetic profile after chronic administration during the whole gestation, with a marked accumulation in fat tissues and mammary glands.

The other main objective of our PBPK model in rat was to predict the internal dose in the fetal brain to help in risk assessment. PBPK models allow inter-species extrapolations (73) and can be used to estimate human fetal exposure in inaccessible compartments such as the brain (74). The estimated concentrations in the fetal brain could aid in the selection of appropriate doses to investigate the neurodevelopmental toxicity using human *in vitro* systems. To assess exposure in the fetal brain and to facilitate its use in a risk assessment context, a compartmental structure was established for the fetal PBPK model, with mass communication via the blood–placenta barrier. Using estimated parameters in pregnant rats and fetuses, the model was able to reproduce correctly the kinetics of both *cis*- and *trans*-isomers in fetal blood and tissues even if *trans*-permethrin was only quantifiable in fetal brain due to analytical limitations. The fetal brain was exposed to permethrin, demonstrating that exposure during pregnancy is of concern for the developing brain even if fetal exposure was less than maternal exposure with a feto/maternal (FM) ratio of 0.54. In blood, the FM ratio was 0.25 for *cis*-permethrin, which is close to the FM ratio of 0.5 reported for cypermethrin, another pyrethroid in placental perfusion studies in humans (75). In our model, transfers from the placenta to the fetuses were considered as a simple diffusion with an estimated placental rate constant value from the dam to the fetuses of 1.91 L/h/kg^0.75^. This value was close to that reported for other pesticides, such as atrazine in rats (76). However, the placental transfer of permethrin could also involve active transports, and further data are needed to characterize placental transfer.

In conclusion, we developed a gestation PBPK model in rats, allowing the identification of key parameters affecting maternal exposure to *cis*- and *trans*-permethrin during gestation and an accurate prediction of fetal brain tissue distribution in rats. In rodents, permethrin and other pyrethroids have shown neurodevelopmental effects. The interpretation of animal studies is challenging due to the lack of assessment of the internal exposure and variable exposure periods and doses used. This model could be used to predict brain levels in reported studies in animals during gestational exposure to aid in risk assessment. Moreover, the mechanisms by which chronic early-life permethrin and pyrethroids could exert developmental neurotoxicity was not well-understood (77). The model allows the estimation of relevant concentrations in the fetal brain in rats. The model could be extrapolated to humans by including specific human values for parameters required for the PBPK model and relevant *in vitro* data for clearance-specific parameters (78). The human pregnancy PBPK model could then be used to estimate relevant concentrations in the fetal brain to test in *in vitro* systems. Once more data will be available, these data could be integrated in specific adverse outcome pathways to assess developmental neurotoxicity in humans.

## Data Availability Statement

The raw data supporting the conclusions of this article will be made available by the authors, without undue reservation.

## Ethics Statement

The animal study was reviewed and approved by a regional Ethic Committee on experiments using animals (CREMEAP no.96) and the French Ministry of Research with the permit number 01812.01.

## Author Contributions

SP and FZ formulated the research questions and designed the studies. SD, FR, and AL performed the experimental study in rats. SP, PM, and AC performed the chemical analysis. SP, FZ, and CB performed the PBPK modeling. CB ran the sensitivity analyses. SP wrote the manuscript. FZ and CB provided critical review and comments. FZ and VB were the supervisors of this research. All authors contributed to the article and approved the submitted version.

## Funding

This work was supported by the French Ministry of Ecology and Sustainable Development (Program 190) and by the HBM4EU project.

## Conflict of Interest

The authors declare that the research was conducted in the absence of any commercial or financial relationships that could be construed as a potential conflict of interest.

## Publisher's Note

All claims expressed in this article are solely those of the authors and do not necessarily represent those of their affiliated organizations, or those of the publisher, the editors and the reviewers. Any product that may be evaluated in this article, or claim that may be made by its manufacturer, is not guaranteed or endorsed by the publisher.
