# Supplementary material for: Evaluation of Placental Transfer and Tissue Distribution of cis- and Trans-Permethrin in Pregnant Rats and Fetuses Using a Physiological-Based Pharmacokinetic Model
Source: Front Pediatr. 2021 Sep 23;9:730383. doi: 10.3389/fped.2021.730383 (PMC8495120; doi:10.3389/fped.2021.730383)
Supplement: Supplementary file 1 [file Data_Sheet_1.docx]

# Tables

**Table S1:** Equations describing growth and changing parameters in pregnant rats and fetuses during the whole gestation period

| **Parameters** | **Equation** | | **References** |
| --- | --- | --- | --- |
| Pregnant rats |  |  |  |
| Tissue volumes (L) | | | |
| Mammary gland  (VMam) | VMam = VMam0 * (1 +(0.201 * GD)) | | Yoon et al., 2009 |
| Fat (VFat) | VFat = VFat0 * (1+(0.044 * GD)) | | Yoon et al., 2009 |
| Placenta (VPla_1F) | (0.6 / (1 +(5000* e ^(-0.0240*GD*24)^))) /1000 | | Emond et al., 2004 modified |
| Body Weight (BW, kg) | BW = BW0 + (VFat - VFat0) + (VMam – VMam0) + N__Fet *_ (V_Pla_1F_ + V_1Fet_) | | Yoon et al., 2009 |
| Cardiac output index  (QCI, L/h/kg) | QCI = 24.56 - (0.1323 * GD) | | Yoon et al., 2009 |
| Total cardiac flow  (QC, L/h/kg) | QC = QCI * BW | | Yoon et al., 2009 |
| Tissue blood flows (L/h) | | | |
| Mammary gland  (QMam) | QMam0 = scF_QMam0 * QC0  QMam = QMam0 * (VMam / VMam0) | | Gentry et al., 2002 |
| Fat (QFat) | QFat0 = scF_QFat0 * QC0  QFat = QFat0 * (VFat / VFat0) | | Gentry et al., 2002 |
| Placenta (QPla_1F) | QPla_1F = ((0.02 * QDEC) + QCAP)/24  where QDEC and QCAP are as follows depending of the GD | | Yoon et al., 2009 |
|  | GD < GD6 | QDEC = 0  QCAP = 0 |  |
|  | GD6 < GD < GD10 | QDEC = 0.55 * (GD - 6)  QCAP = 0 |  |
|  | GD10 < GD < GD12 | QDEC = 2.2 * e ^-0.23 * (GD - 10)^  QCAP = 0 |  |
|  | GD12 < GD | QDEC = 2.2 * e ^-0.23 * (GD - 10)^  QCAP = 0.1207*(GD-12) ^4.36^ |  |
| Fetus | | | |
| Body weight (V1Fet) | V1Fet = 0.1089 + [16 * e^-e (5.515 - (0.2565 * GD))^] / 1000 | | Yoon et al., 2009 |
| Tissue volumes (L) | | | |
| Liver (VLiv_1F) | V_Liv_1F_ = [0.3152 * e^-e (11.49 - (0.649 * GD))^] / 1000 | | Yoon et al., 2009 |
| Brain (VBrain_1F) | V_Brain_1F_ = [4.191 * e^-e (2.554 -(0.06726 * GD))^] 1000 | | Yoon et al., 2009 |

N_Fet = number of fetuses

GD = gestation day

“_0_”: indicates parameter value at GD0

# Sensitivity Analyses

**
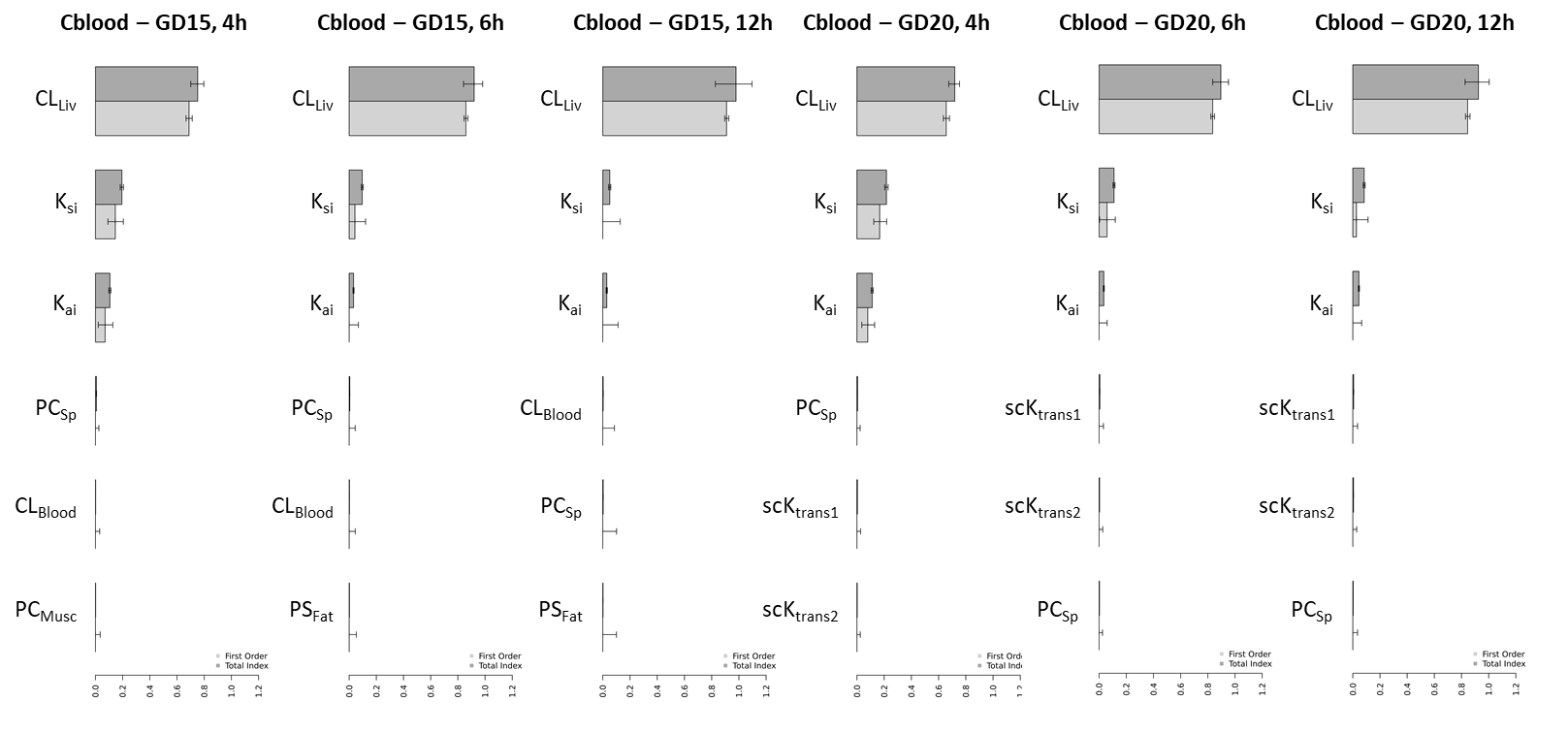
**

**Figure S1:** First (light grey) and total (dark grey) order sensitive indices for the maternal blood concentration at GD15 and GD20 at three time points after the oral administration (4h, 6h and 12h). The abbreviations for the model parameters are given in Table 2.


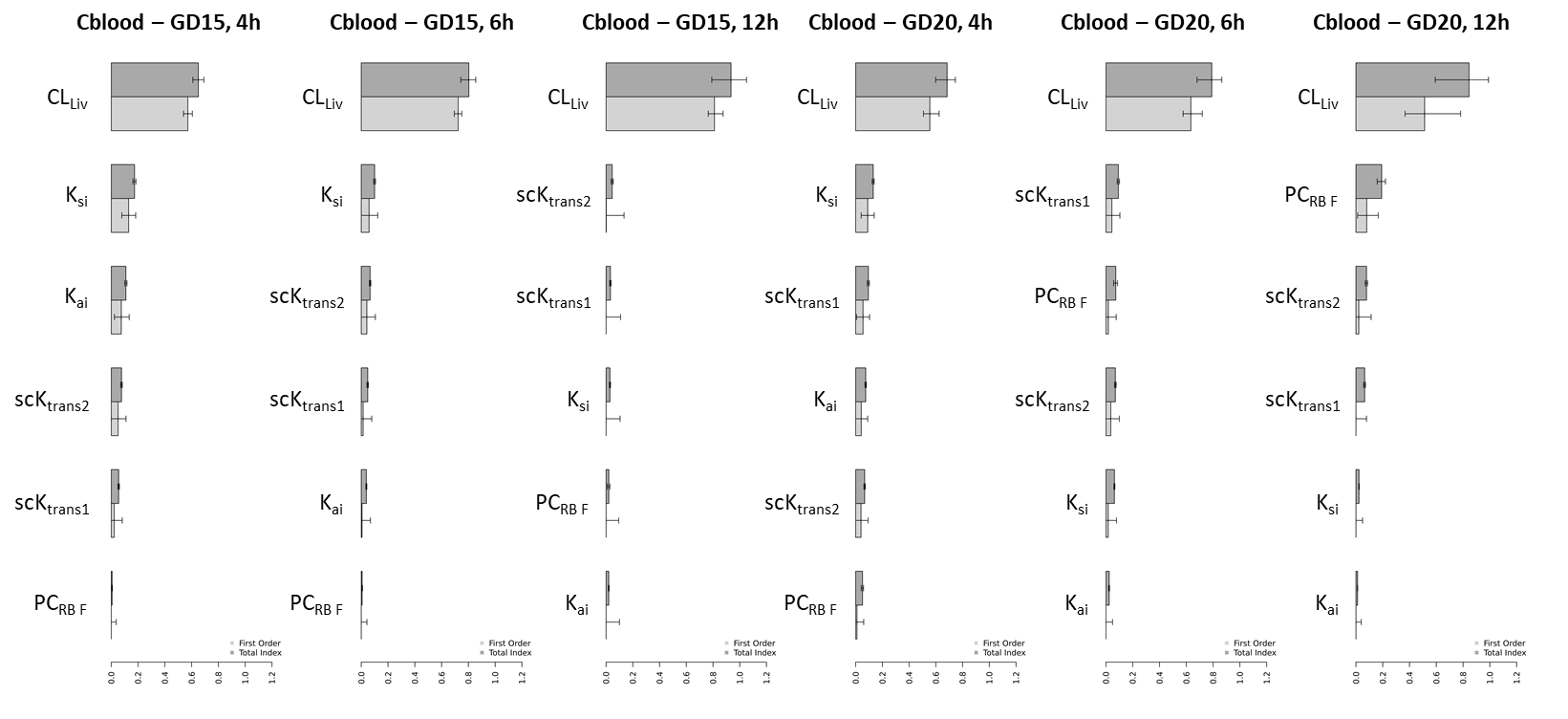


**Figure S2:** First (light grey) and total (dark grey) order sensitive indices for the fetal blood concentration at GD15 and GD20 at three time points after the oral administration (4h, 6h and 12h). The abbreviations for the model parameters are given in Table 2.


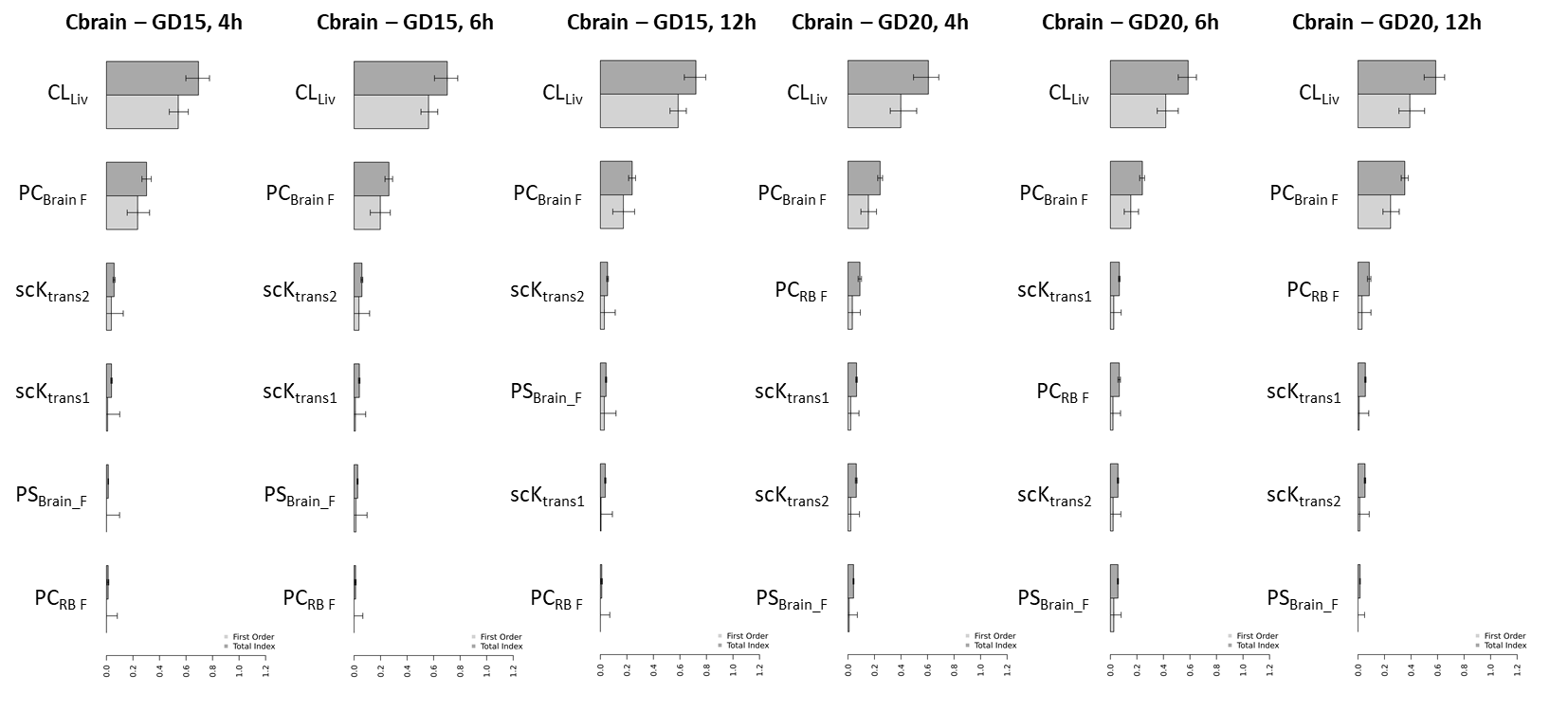


**Figure S3:** First (light grey) and total (dark grey) order sensitive indices for the fetal brain concentration at GD15 and GD20 at three time points after the oral administration (4h, 6h and 12h). The abbreviations for the model parameters are given in Table 2.

# Model code

**#** Model code for rat gestation PBPK model for *cis*- and *trans*-permethrin

**# Units**

# Body weight: kg

# Volumes: L

# Time: h

# Flows: L/h

# Clearance: L/h/kg

# Dose administered: microg/kg/day

# Amount: microg

# Concentration: microg/L

# Rate constant: h-1

#################################################################################

**States = {**

**# Absorption**

ACisPerm_gav, # Amount administered by gavage

ACisPerm_abs, # Amount absorbed

ATransPerm_gav, # Amount administered by gavage

ATransPerm_abs, # Amount absorbed

**# Amount in tissues - dam**

*# Cis-permethrin*

ACisPerm_Blood, # Blood

ACisPerm_GI1, # 1st theorical compartment of the gastrointestinal tract

ACisPerm_GI2, # 2nd theorical compartment of the gastrointestinal tract

ACisPerm_GI, # Gastro-intestinal tract

ACisPerm_Liv, # Liver

ACisPerm_Rp, # Rapidly perfused tissues

ACisPerm_Pla, # Placenta

ACisPerm_Ext_Kid, # Extracellular kidney space

ACisPerm_Int_Kid, # Intracellular kidney space

ACisPerm_Ext_Fat, # Extracellular fat space

ACisPerm_Int_Fat, # Intracellular fat space

ACisPerm_Ext_Mam, # Extracellular mammary glands space

ACisPerm_Int_Mam, # Intracellular mammary glands space

ACisPerm_Ext_Brain, # Extracellular brain space

ACisPerm_Int_Brain, # Intracellular brain space

ACisPerm_Ext_Sp, # Extracellular slowly perfused space

ACisPerm_Int_Sp, # Intracellular slowly perfused space

ACisPerm_Ext_Musc, # Extracellular muscle space

ACisPerm_Int_Musc, # Intracellular muscle space

*# Trans-permethrin*

ATransPerm_Blood, # Blood

ATransPerm_GI1, # 1st theorical compartment of the gastrointestinal tract

ATransPerm_GI2, # 2nd theorical compartment of the gastrointestinal tract

ATransPerm_GI, # Gastro-intestinal tract

ATransPerm_Liv, # Liver

ATransPerm_Rp, # Rapidly perfused tissues

ATransPerm_Pla, # Placenta

ATransPerm_Ext_Kid, # Extracellular kidney space

ATransPerm_Int_Kid, # Intracellular kidney space

ATransPerm_Ext_Fat, # Extracellular fat space

ATransPerm_Int_Fat, # Intracellular fat space

ATransPerm_Ext_Mam, # Extracellular mammary glands space

ATransPerm_Int_Mam, # Intracellular mammary glands space

ATransPerm_Ext_Brain, # Extracellular brain space

ATransPerm_Int_Brain, # Intracellular brain space

ATransPerm_Ext_Sp, # Extracellular slowly perfused space

ATransPerm_Int_Sp, # Intracellular slowly perfused space

ATransPerm_Ext_Musc, # Extracellular muscle space

ATransPerm_Int_Musc, # Intracellular muscle space

**# Amount metabolized - dam**

A_CisPerm_metabo,

A_TransPerm_metabo,

**# Amount excreted in feces - dam**

A_CisPerm_Fec,

A_TransPerm_Fec,

**# Amount eliminated (metabolism + excretion) - dam**

A_CisPerm_Elim,

A_TransPerm_Elim,

**# Amount metabolized - fetus**

A_CisPerm_metabo_F,

A_TransPerm_metabo_F,

**# Amount eliminated (metabolism + excretion) - fetus**

A_CisPerm_Elim_F,

A_TransPerm_Elim_F,

**# Foeto-maternal Transferts**

*# Cis-permethrin*

A_CisPerm_Trans_Fet,

A_CisPerm_Trans_Pla,

*#Trans-permethrin*

A_TransPerm_Trans_Fet,

A_TransPerm_Trans_Pla,

**# Amount in tissues - fetus**

*# Cis-permethrin*

ACisPerm_Blood_F, # Fetal blood

ACisPerm_Liv_F, # Fetal liver

A_CisPerm_RB_F, # Rest of the fetal body

A_CisPerm_Int_Brain_F, # Intracellular brain space of the fetus

A_CisPerm_Ext_Brain_F, # Extracellular brain space of the fetus

*# Trans-permethrin*

ATransPerm_Blood_F,  # Fetal blood

ATransPerm_Liv_F, # Fetal liver

A_TransPerm_RB_F, # Rest of the fetal body

A_TransPerm_Int_Brain_F, # Intracellular brain space of the fetus

A_TransPerm_Ext_Brain_F, # Extracellular brain space of the fetus

};

#################################################################################

**Outputs = {**

dA_CisPerm_Fec,

dA_CisPerm_Fec_old,

dA_TransPerm_Fec,

dA_TransPerm_Fec_old,

t_old,

GD, # Gestation day

BW, # Body weight of the dam

**#Tissue volumes**

VFat, # Fat

VMam, # Mammary glands

VPla_1F, # Placenta for one single fetus

VPla, # Placentas for the whole litter

V1Fet, # One single fetus

VFet, # Fetuses for the whole litter

VLiv_1F, # Liver for one single fetus

VLiv_F, # Liver for the whole litter

VBrain_1F, # Brain for one single fetus

VBrain_F, # Brain for the whole litter

VBlood_F, # Blood

VRB_F, # Rest of the fetal body

**#Tissue blood flows**

QC, # Cardiac output

QFat, # Fat

QMam, # Mammary glands

QRp, # Rapidly perfused tissues

QDEC, #

QCAP, #

QPla_1F, # Placenta for one single fetus

QPla, # Placenta for the whole litter

QC_1F, # Fetal cardiac output for one single fetus

QC_F, # Fetal cardiac output for the whole litter

QLiv_F, # Liver

QBrain_F, # Brain

QRB_F, # Rest of the fetal body

**# Check mass balance**

*# Cis-permethrin*

ACisPerm_Tissue,

ACisPerm_Total,

ACisPerm_Check,

ACisPerm_Tissue_F,

ACisPerm_Check_F,

*# Trans-permethrin*

ATransPerm_Tissue,

ATransPerm_Total,

ATransPerm_Check,

ATransPerm_Tissue_F,

ATransPerm_Check_F,

**# Blood and tissue concentrations - dam**

*# Cis-permethrin*

C_CisPerm_Blood, # Blood

C_CisPerm_Liv, # Liver

C_CisPerm_Musc, # Muscle

C_CisPerm_Kid, # Kidney

C_CisPerm_Pla, # Placenta

C_CisPerm_Fat, # Fat

C_CisPerm_Mam, # Mammary glands

C_CisPerm_Brain, # Brain

C_CisPerm_Sp, # Slowly perfused tissues

C_CisPerm_Rp, # Rapidly perfused tissues

*# Trans-permethrin*

C_TransPerm_Blood, # Blood

C_TransPerm_Liv, # Liver

C_TransPerm_Musc, # Muscle

C_TransPerm_Kid, # Kidney

C_TransPerm_Pla, # Placenta

C_TransPerm_Fat, # Fat

C_TransPerm_Mam, # Mammary glands

C_TransPerm_Brain, # Brain

C_TransPerm_Sp, # Slowly perfused tissues

C_TransPerm_Rp, # Rapidly perfused tissues

**# Blood and tissue concentrations - fetus**

*# Cis-permethrin*

C_CisPerm_Blood_F, # Fetal blood

C_CisPerm_Brain_F, # Fetal brain

C_CisPerm_Liv_F, # Fetal liver

C_CisPerm_RB_F, # Rest of the fetal body

*# Trans-permethrin*

C_TransPerm_Blood_F, # Fetal blood

C_TransPerm_Brain_F, # Fetal brain

C_TransPerm_Liv_F, # Fetal liver

C_TransPerm_RB_F, # Rest of the fetal body

};

#################################################################################

**Inputs =** {

DCisPerm_gav, # Quantity of cis-permethrin administered by gavage (microg/kg/day)

DTransPerm_gav, # Quantity of trans-permethrin administered by gavage (microgg/kg/day)

BW0, # Body weight at gestational day (GD) 0

N_Fet, # Number of fetuses by litter

t_voiding, # Time of elimination

};

#################################################################################

**# Physiological and Pharmacokinetic Parameters - Dam**

################################################

**# Cardiac index at GD0**

QCI0;

**# Fractional tissue volumes**

scF_VGI; # Gastro intestinal

scF_VLiv; # Liver

scF_VMusc; # Muscles

scF_VBrain; # Brain

scF_VRp; # Rapidly perfused tissues

scF_VKid; # Kidneys

scF_VFat0; # Fat at GD0

scF_VMam0; # Mammary glands at GD0

scF_VBlood; # Blood

scF_V_Ext_Kid; # Kidney

scF_V_Ext_Fat; # Fat

scF_V_Ext_Mam; # Mammary glands

scF_V_Ext_Brain; # Brain

scF_V_Ext_Sp; # Slowly perfused tissues

scF_V_Ext_Musc; # Muscles

scF_VNP; # Non perfused tissues

**# Fractional tissue blood flow (fraction of cardiac output)**

scF_QGI; # Hepatic portal vein

scF_QLiv; # Hepatic artery blood flow fraction

scF_QMusc; # Muscles

scF_QBrain; # Brain

scF_QSp; # Slowly perfused tissues

scF_QKid; # Kidneys

scF_QFat0; # Fat at GD0

scF_QMam0; # Mammary glands at GD0

**# Rate constants (h-1)**

*# Cis-permethrin*

KsI_CisPerm; # Rate of transfer between GI compartments

KaI_CisPerm; # Absorption rate constant from theorical intestinal

KFec_CisPerm; # Fecal excretion rate constant

*# Trans-permethrin*

KsI_TransPerm; # Rate of transfer between GI compartments

KaI_TransPerm; # Absorption rate constant from theorical intestinal

KFec_TransPerm; # Fecal excretion rate constant

**# Tissue/blood partition coefficients (PC)**

*# Cis-permethrin*

PC_Liv_CisPerm; # Liver: blood PC

PC_GI_CisPerm; # GI: blood PC

PC_Fat_CisPerm;  # Fat: blood PC

PC_Mam_CisPerm; # Mammary gland: blood PC

PC_Musc_CisPerm; # Muscle: blood PC

PC_Brain_CisPerm; # Brain: blood PC

PC_Sp_CisPerm; # Slowly perfused tissues: blood PC

PC_Rp_CisPerm; # Rapidly perfused tissues: blood PC

PC_Kid_CisPerm; # Kidney: blood PC

PC_Pla_CisPerm; # Placenta: blood PC

*# Trans-permethrin*

PC_Liv_TransPerm; # Liver: blood PC

PC_GI_TransPerm; # GI: blood PC

PC_Fat_TransPerm; # Fat: blood PC

PC_Mam_TransPerm; # Mammary gland: blood PC

PC_Musc_TransPerm; # Muscle: blood PC

PC_Brain_TransPerm; # Brain: blood PC

PC_Sp_TransPerm; # Slowly perfused tissues: blood PC

PC_Rp_TransPerm; # Rapidly perfused tissues: blood PC

PC_Kid_TransPerm; # Kidney: blood PC

PC_Pla_TransPerm; # Placenta: blood PC

**# Permeability coefficients (fraction of tissue flow L/h)**

scF_PS_Kid; # Kidneys

scF_PS_Fat_CisPerm; # Fat

scF_PS_Fat_TransPerm; # Fat

scF_PS_Mam_CisPerm; # Mammary glands

scF_PS_Mam_TransPerm; # Mammary glands

scF_PS_Brain_CisPerm; # Brain

scF_PS_Brain_TransPerm; # Brain

scF_PS_Sp_CisPerm; # Slowly perfused tissues

scF_PS_Sp_TransPerm; # Slowly perfused tissues

scF_PS_Musc_CisPerm; # Muscles

scF_PS_Musc_TransPerm; # Muscles

**# Metabolic Clearances**

*# Cis-permethrin*

sc_CL_CisPerm_GI; # Gastro-intestinal

sc_CL_CisPerm_Blood; # Blood

sc_CL_CisPerm_Liv; # Liver

*# Trans-permethrin*

sc_CL_TransPerm_GI; # Gastro-intestinal

sc_CL_TransPerm_Blood; # Blood

sc_CL_TransPerm_Liv; # Liver

**# Foeto-placental transferts (L/h/kg 0.75)**

scKTrans1; # sclaled rate of transfer from placenta to fetal venous blood

*#* scKTrans2; # sclaled rate of transfer from fetal venous blood to placenta

Ratio_scKTrans; # (= scKTrans1/ scKTrans2);

**# Physiological and Pharmacokinetic Parameters - Fetuses**

##################################################

**# Fractional tissue volumes - fetus**

scF_VBlood_F; # Blood

**# Cardiac index - fetus**

QCI_1F;

**# Fractional tissue blood flows - fetus**

scF_QBrain_F; # Fetal brain

scF_QLiv_F; # Fetal liver

**# Fractional tissue volumes**

scF_V_Ext_Brain_F_PERM; # Fetal brain

**# Tissue/blood partition coefficients - fetus**

PC_Brain_F; # Brain: blood PC

PC_Liv_F; # Liver: blood PC

PC_RB_F; # Rest of the body: blood PC

**# Metabolic clearances - fetus**

*# Cis-permethrin*

sc_CL_CisPerm_Blood_F; # Blood

sc_CL_CisPerm_Liv_F; # Liver

*# Trans-permethrin*

sc_CL_TransPerm_Blood_F; # Blood

sc_CL_TransPerm_Liv_F; # Liver

**# Permeability coefficients (fraction of tissue flow L/h) - fetus**

scF_PS_Brain_F; # Brain

**# Scaled parameters**

####################

VFat0; # Fat volume at GD0

VMam0; # Mammary gland volume at GD0

VGI; # Gastro-intestinal tract volume

VLiv; # Liver volume

VRp; # Rapidly perfused tissues volume

VKid; # Kidney volume

V_Ext_Kid;  # Extracellular volume of the kidney

V_Int_Kid; # Intracellular volume of the kidney

VBlood; # Blood volume

VNP; # Non-perfused tissues

VMusc; # Muscles volume

V_Ext_Musc; # Extracellular volume of the muscle

V_Int_Musc; # Intracellular volume of the muscle

VSp; # Slowly perfused tissues volume

V_Ext_Sp; # Extracellular volume of the slowly perfused tissues

V_Int_Sp; # Intracellular volume of the slowly perfused tissues

VBrain; # Brain volume

V_Ext_Brain;# Extracellular volume of the brain

V_Int_Brain; # Intracellular volume of the brain

QC0; # QC at GD0

QGI; # Gastro-intestinal blood flow

QLiv; # Liver blood flow

QLiv_out;

QMusc; # Muscle blood flow

QBrain; # Brain blood flow

QSp; # Slowly perfused tssues blood flow

QKid; # Kidney blood flow

QMam0; # Mammary glands blood flow at GD0

QFat0; # Fat blood flow at GD0

PS_Kid; # Kidney

PS_Brain_CisPerm; # Brain

PS_Brain_TransPerm; # Brain

PS_Sp_CisPerm; # Slowly perfused

PS_Sp_TransPerm; # Slowly perfused

PS_Musc_CisPerm; # Muscles

PS_Musc_TransPerm; # Muscles

scKTrans2; # sclaled rate of transfert from fetal to placenta

#################################################################################

**Scale {**

**# Tissue volumes at GD0 for changing tissue volume during gestation**

VFat0 = scF_VFat0 * BW0;

VMam0 = scF_VMam0 * BW0;

**# Tissue volume with a constant volume**

VGI = BW0 * scF_VGI;

VLiv = BW0 * scF_VLiv;

VRp = BW0 * scF_VRp;

VKid = BW0 * scF_VKid;

V_Ext_Kid = VKid * scF_V_Ext_Kid;

V_Int_Kid = VKid * (1 - scF_V_Ext_Kid);

VBlood = BW0 * scF_VBlood;

VNP = BW0 * scF_VNP;

VMusc = BW0 * scF_VMusc;

V_Ext_Musc = VMusc * scF_V_Ext_Musc;

V_Int_Musc = VMusc * (1 - scF_V_Ext_Musc);

VBrain = BW0 * scF_VBrain;

V_Ext_Brain = VBrain * scF_V_Ext_Brain;

V_Int_Brain = VBrain * (1 - scF_V_Ext_Brain);

VSp = BW0 * (1 - scF_VGI -scF_VLiv - scF_VMusc - scF_VBrain - scF_VRp - scF_VKid

- scF_VBlood - scF_VFat0 -scF_VMam0 - scF_VNP);

V_Ext_Sp = VSp * scF_V_Ext_Sp;

V_Int_Sp = VSp * (1 - scF_V_Ext_Sp);

**# Cardiac output at GD0**

QC0 = QCI0 * BW0;

**# Blood flows constant during gestation**

QGI = QC0 * scF_QGI;

QLiv = QC0 * scF_QLiv;

QLiv_out = QGI + QLiv;

QMusc = QC0 * scF_QMusc;

QBrain = QC0 * scF_QBrain;

QSp = QC0 * scF_QSp;

QKid = QC0 * scF_QKid;

**# Permeability coefficients**

PS_Kid = scF_PS_Kid * QKid;

PS_Brain_CisPerm = scF_PS_Brain_CisPerm * QBrain;

PS_Brain_TransPerm = scF_PS_Brain_TransPerm * QBrain;

PS_Sp_CisPerm = scF_PS_Sp_CisPerm * QSp;

PS_Sp_TransPerm = scF_PS_Sp_TransPerm * QSp;

PS_Musc_CisPerm = scF_PS_Musc_CisPerm * QMusc;

PS_Musc_TransPerm = scF_PS_Musc_TransPerm * QMusc;

**# Tissue blood flows at GD0 for changing tissue blood flows during gestation**

QMam0 = scF_QMam0 * QC0;

QFat0 = scF_QFat0 * QC0;

**# Rate transfer from fetal blood to placenta**

scKTrans2 = scKTrans1 / Ratio_scKTrans;

}; # End of model scaling

#################################################################################

**Dynamics {**

**# Gestation day (GD)**

GD = t / 24;

**# Dam**

#######

**# Changing tissue volumes**

*# Volume of mammary glands (L)*

VMam = VMam0 * (1 +(0.201 * GD));

V_Ext_Mam = VMam * scF_V_Ext_Mam;

V_Int_Mam = VMam * (1 - scF_V_Ext_Mam);

*# Volume of fat (L)*

VFat = VFat0 * (1+(0.044 * GD));

V_Ext_Fat = VFat * scF_V_Ext_Fat;

V_Int_Fat = VFat * (1 - scF_V_Ext_Fat);

*# Volume of placenta for one fetus (L)*

VPla_1F = (0.6 / (1 +(5000* exp(-0.0240*GD*24))))/1000;

*# Volume of placenta for a whole litter (L)*

VPla = VPla_1F * N_Fet;

**# Body weight - dam**

BW = BW0 + (VFat - VFat0) + (VMam -VMam0) + VPla + VFet;

**# Cardiac output - dam**

QCI = 24.56 - (0.1323 * GD);

QC = QCI * BW;

**# Changing blood flows - dam**

*# Blood flow to mammary glands*

QMam = QMam0 * (VMam / VMam0);

*# Blood flow to fat*

QFat = QFat0 * (VFat / VFat0);

*# BLood flow to QDEC for one fetus*

QDEC = (GD< 6 ? 0.0 :

(GD < 10 ? 0.55 * (GD - 6) : 2.2 * exp(-0.23 * (GD - 10))));

*# Blood flow to QCAP for one fetus*

QCAP = (GD < 12 ? 0.0 : pow((0.1207 * (GD - 12)), 4.36));

*# Blood flow to placenta for one fetus (L/h)*

QPla_1F = ((0.02 * QDEC) + QCAP)/24;

*# Blood flow to placenta for a whole litter (L/h)*

QPla = QPla_1F * N_Fet;

*# Blood flow to Rp*

QRp = QC - QMam - QFat - QGI - QLiv - QMusc - QBrain - QSp - QKid - QPla;

**# Permeability coefficients - dam**

PS_Fat_CisPerm = scF_PS_Fat_CisPerm * QFat;

PS_Fat_TransPerm = scF_PS_Fat_TransPerm * QFat;

PS_Mam_CisPerm = scF_PS_Mam_CisPerm * QMam;

PS_Mam_TransPerm = scF_PS_Mam_TransPerm * QMam;

**# Metabolic Clearances - dam**

*# Cis-permethrin*

CL_CisPerm_GI = sc_CL_CisPerm_GI * BW;

CL_CisPerm_Blood = sc_CL_CisPerm_Blood * BW;

CL_CisPerm_Liv = sc_CL_CisPerm_Liv * BW;

*# Trans-permethrin*

CL_TransPerm_GI = sc_CL_TransPerm_GI * BW;

CL_TransPerm_Blood = sc_CL_TransPerm_Blood * BW;

CL_TransPerm_Liv = sc_CL_TransPerm_Liv * BW;

**# Fetus**

#######

**# Changing tissue volumes - fetus**

*# Tissue volume for one fetus (L)*

V1Fet = (0.1089 + (16 * exp(-exp(5.515-(0.2565 * GD))))) / 1000;

*# Tissue volume for a whole litter (L)*

VFet = V1Fet * N_Fet;

*# Volume of brain for a whole litter (L)*

VBrain_1F = (4.191 * exp(-exp(2.554 -(0.06726 * GD)))) / 1000;

VBrain_F = VBrain_1F * N_Fet;

V_Ext_Brain_F = VBrain_F * scF_V_Ext_Brain_F_PERM;

V_Int_Brain_F = VBrain_F * (1 - scF_V_Ext_Brain_F_PERM);

*# Volume of liver for a whole litter (L)*

VLiv_1F = (0.3152 * exp(-exp(11.49 - (0.649 * GD)))) / 1000;

VLiv_1F_16 = (0.3152 * exp(-exp(11.49 - (0.649 * 16)))) / 1000;

VLiv_F = (GD < 16 ? (VLiv_1F_16 * N_Fet) : (VLiv_1F * N_Fet));

*# Volume of blood for a whole litter (L)*

VBlood_F = VFet * scF_VBlood_F;

*# Volume of the rest of the body for a whole litter (L)*

VRB_F = VFet - (VBrain_F + VLiv_F + VBlood_F);

**# Permeability coefficients - fetus**

PS_Brain_F_CisPerm = scF_PS_Brain_F * QBrain_F;

PS_Brain_F_TransPerm = scF_PS_Brain_F * QBrain_F;

**# Cardiac output - fetus (L/h)**

QC_1F = QCI_1F * V1Fet; # for one fetus

QC_F = QC_1F * N_Fet;

**# Changing blood flows - fetus**

*# Blood flow to brain for the whole litter (L/h)*

QBrain_F = QC_F * scF_QBrain_F;

*# Blood flow to liver for the whole litter (L/h)*

QLiv_F = QC_F * scF_QLiv_F;

*# Blood flow to the rest of the body for the whole litter (L/h)*

QRB_F = QC_F - QBrain_F - QLiv_F;

**# Metabolic Clearances - fetus**

*# Cis-permethrin*

CL_CisPerm_Blood_F = sc_CL_CisPerm_Blood_F *(pow(V1Fet,0.75)*N_Fet) ;

CL_CisPerm_Liv_F = sc_CL_CisPerm_Liv_F * (pow(V1Fet,0.75)*N_Fet);

*# Trans-permethrin*

CL_TransPerm_Blood_F = sc_CL_TransPerm_Blood_F * (pow(V1Fet,0.75)*N_Fet);

CL_TransPerm_Liv_F = sc_CL_TransPerm_Liv_F * (pow(V1Fet,0.75)*N_Fet);

**# Materno-foetal transfers - (L/h)**

KTrans1 = scKTrans1 * (pow(V1Fet,0.75)*N_Fet);

KTrans2 = scKTrans2 * (pow(V1Fet,0.75)*N_Fet);

**# Dynamics – *cis*-permethrin**

############################

**# Concentrations - dam**

*# Extracellular (venous) tissue space concentrations for diffusion limited compartments*

C_CisPerm_Ext_Kid = ACisPerm_Ext_Kid / V_Ext_Kid;

C_CisPerm_Ext_Fat = ACisPerm_Ext_Fat / V_Ext_Fat;

C_CisPerm_Ext_Mam = ACisPerm_Ext_Mam / V_Ext_Mam;

C_CisPerm_Ext_Brain = ACisPerm_Ext_Brain / V_Ext_Brain;

C_CisPerm_Ext_Sp = ACisPerm_Ext_Sp / V_Ext_Sp;

C_CisPerm_Ext_Musc = ACisPerm_Ext_Musc / V_Ext_Musc;

*# Intracellular (tissue) concentrations for diffusion limited compartments*

C_CisPerm_Int_Kid = ACisPerm_Int_Kid / V_Int_Kid;

C_CisPerm_Int_Fat = ACisPerm_Int_Fat / V_Int_Fat;

C_CisPerm_Int_Mam = ACisPerm_Int_Mam / V_Int_Mam;

C_CisPerm_Int_Brain = ACisPerm_Int_Brain / V_Int_Brain;

C_CisPerm_Int_Sp = ACisPerm_Int_Sp / V_Int_Sp;

C_CisPerm_Int_Musc = ACisPerm_Int_Musc / V_Int_Musc;

*# Tissue concentrations for GI and Liver*

#C_CisPerm_GI = ACisPerm_GI / VGI;

#C_CisPerm_Liv = ACisPerm_Liv / VLiv;

*# Concentrations (Cout) at organ exit*

# flow-limited compartments

Cout_CisPerm_GI = ACisPerm_GI / (VGI * PC_GI_CisPerm);

Cout_CisPerm_Liv = ACisPerm_Liv / (VLiv * PC_Liv_CisPerm);

Cout_CisPerm_Rp = ACisPerm_Rp / (VRp * PC_Rp_CisPerm);

Cout_CisPerm_Pla = ACisPerm_Pla / (VPla * PC_Pla_CisPerm);

# diffusion limited compartments

Cout_CisPerm_Kid = C_CisPerm_Int_Kid / PC_Kid_CisPerm;

Cout_CisPerm_Fat = C_CisPerm_Int_Fat / PC_Fat_CisPerm;

Cout_CisPerm_Mam = C_CisPerm_Int_Mam / PC_Mam_CisPerm;

Cout_CisPerm_Brain = C_CisPerm_Int_Brain / PC_Brain_CisPerm;

Cout_CisPerm_Sp = C_CisPerm_Int_Sp / PC_Sp_CisPerm;

Cout_CisPerm_Musc = C_CisPerm_Int_Musc / PC_Musc_CisPerm;

*# Arterial blood concentration*

Ca_CisPerm = ACisPerm_Blood / VBlood;

**# Concentrations – fetus (whole litter)**

*# Extracellular (venous) tissue space concentrations for diffusion limited compartments*

C_CisPerm_Ext_Brain_F = A_CisPerm_Ext_Brain_F / V_Ext_Brain_F;

*# Intracellular (tissue) concentrations for diffusion limited cpts*

C_CisPerm_Int_Brain_F = A_CisPerm_Int_Brain_F / V_Int_Brain_F;

*# Tissue concentration for liver*

C_CisPerm_Liv_F = ACisPerm_Liv_F / VLiv_F;

*# Tissue concentration for rest of the body*

C_CisPerm_RB_F = A_CisPerm_RB_F / VRB_F;

*# Venous blood concentrations at organ exit*

# Diffusion limited compartments

Cout_CisPerm_Brain_F = C_CisPerm_Int_Brain_F / PC_Brain_F;

# Flow limited compartments

Cout_CisPerm_Liv_F = ACisPerm_Liv_F / (VLiv_F * PC_Liv_F);

Cout_CisPerm_RB_F = A_CisPerm_RB_F / PC_RB_F;

*# Arterial blood concentration*

Ca_CisPerm_F = ACisPerm_Blood_F / VBlood_F;

**# Rate of absorption**

dt(ACisPerm_gav) = DCisPerm_gav * BW;

dt(ACisPerm_GI1) = (DCisPerm_gav * BW)-(KsI_CisPerm * ACisPerm_GI1);

dt(ACisPerm_GI2) = (KsI_CisPerm * ACisPerm_GI1) - (KaI_CisPerm * ACisPerm_GI2) -

(KFec_CisPerm * ACisPerm_GI2);

dt(ACisPerm_abs) = KaI_CisPerm * ACisPerm_GI2;

**# Rate of metabolism - dam**

r_CisPerm_metabo_GI = CL_CisPerm_GI * Cout_CisPerm_GI;

r_CisPerm_metabo_Blood = CL_CisPerm_Blood * Ca_CisPerm;

r_CisPerm_metabo_Liv = CL_CisPerm_Liv * Cout_CisPerm_Liv;

**# Rate of formation of metabolites - dam**

dt(A_CisPerm_metabo) = r_CisPerm_metabo_GI + r_CisPerm_metabo_Blood

+ r_CisPerm_metabo_Liv;

**# Rate of excretion - dam**

dt(A_CisPerm_Fec) = KFec_CisPerm * ACisPerm_GI2;

dA_CisPerm_Fec_old = ((t_voiding!=0) ? A_CisPerm_Fec : dA_CisPerm_Fec_old);

t_old = (t_voiding!=0?t:t_old);

dA_CisPerm_Fec = ((t-t_old)>0 ? A_CisPerm_Fec-dA_CisPerm_Fec_old : dA_CisPerm_Fec );

**# Rate of placenta to fetus transfer**

r_CisPerm_to_foetus = KTrans1 * Cout_CisPerm_Pla;

dt(A_CisPerm_Trans_Fet) = KTrans1 * Cout_CisPerm_Pla;

**# Rate of fetus to placenta transfer**

r_CisPerm_to_placenta = KTrans2 * Ca_CisPerm_F;

dt(A_CisPerm_Trans_Pla) = KTrans2 * Ca_CisPerm_F;

**# Rate of elimination - dam**

dt(A_CisPerm_Elim) = dt(A_CisPerm_metabo) + dt(A_CisPerm_Fec) + dt(A_CisPerm_Trans_Fet) - dt(A_CisPerm_Trans_Pla);

**# Differential for amounts in tissues - dam**

# Flow-limited compartments without metabolism

dt(ACisPerm_Rp) = QRp * (Ca_CisPerm - Cout_CisPerm_Rp);

# Diffusion limited compartments

*# Kidney*

dt(ACisPerm_Int_Kid) = PS_Kid * (C_CisPerm_Ext_Kid - Cout_CisPerm_Kid);

dt(ACisPerm_Ext_Kid) = QKid * (Ca_CisPerm - C_CisPerm_Ext_Kid)

+ PS_Kid * (Cout_CisPerm_Kid - C_CisPerm_Ext_Kid);

*# Fat*

dt(ACisPerm_Int_Fat) = PS_Fat_CisPerm * (C_CisPerm_Ext_Fat - Cout_CisPerm_Fat);

dt(ACisPerm_Ext_Fat) = QFat * (Ca_CisPerm - C_CisPerm_Ext_Fat)

+ PS_Fat_CisPerm * (Cout_CisPerm_Fat - C_CisPerm_Ext_Fat);

*# Mam*

dt(ACisPerm_Int_Mam) = PS_Mam_CisPerm * (C_CisPerm_Ext_Mam - Cout_CisPerm_Mam);

dt(ACisPerm_Ext_Mam) = QMam * (Ca_CisPerm - C_CisPerm_Ext_Mam)

+ PS_Mam_CisPerm * (Cout_CisPerm_Mam - C_CisPerm_Ext_Mam);

*# Brain*

dt(ACisPerm_Int_Brain) = PS_Brain_CisPerm * (C_CisPerm_Ext_Brain - Cout_CisPerm_Brain);

dt(ACisPerm_Ext_Brain) = QBrain * (Ca_CisPerm - C_CisPerm_Ext_Brain)

+ PS_Brain_CisPerm * (Cout_CisPerm_Brain - C_CisPerm_Ext_Brain);

*# Muscles*

dt(ACisPerm_Int_Musc) = PS_Musc_CisPerm * (C_CisPerm_Ext_Musc - Cout_CisPerm_Musc);

dt(ACisPerm_Ext_Musc) = QMusc * (Ca_CisPerm - C_CisPerm_Ext_Musc)

+ PS_Musc_CisPerm * (Cout_CisPerm_Musc - C_CisPerm_Ext_Musc);

*# Sp*

dt(ACisPerm_Int_Sp) = PS_Sp_CisPerm *(C_CisPerm_Ext_Sp - Cout_CisPerm_Sp);

dt(ACisPerm_Ext_Sp) = QSp *(Ca_CisPerm - C_CisPerm_Ext_Sp)

+ PS_Sp_CisPerm *(Cout_CisPerm_Sp - C_CisPerm_Ext_Sp);

# Gastro-intestinal

dt(ACisPerm_GI) = QGI * (Ca_CisPerm - Cout_CisPerm_GI) + dt(ACisPerm_abs)

- r_CisPerm_metabo_GI;

# Liver

dt(ACisPerm_Liv) = (QLiv * Ca_CisPerm) + (QGI * Cout_CisPerm_GI)

- (QLiv_out * Cout_CisPerm_Liv) - r_CisPerm_metabo_Liv;

# Placenta

dt(ACisPerm_Pla) = QPla * (Ca_CisPerm - Cout_CisPerm_Pla) - r_CisPerm_to_foetus

+ r_CisPerm_to_placenta;

**# Venous and arterial blood - dam**

Cv_CisPerm = ((QLiv_out * Cout_CisPerm_Liv)

+ (QMusc * C_CisPerm_Ext_Musc) + (QKid * C_CisPerm_Ext_Kid)

+ (QFat * C_CisPerm_Ext_Fat) + (QMam * C_CisPerm_Ext_Mam)

+ (QBrain * C_CisPerm_Ext_Brain) + (QSp * C_CisPerm_Ext_Sp)

+ (QRp * Cout_CisPerm_Rp)

+ (QPla * Cout_CisPerm_Pla))/QC;

dt(ACisPerm_Blood) = QC * (Cv_CisPerm -Ca_CisPerm) - r_CisPerm_metabo_Blood;

**# Rate of metabolism – fetus**

# Blood

r_CisPerm_metabo_Blood_F = CL_CisPerm_Blood_F * Ca_CisPerm_F;

# Liver

r_CisPerm_metabo_Liv_F = CL_CisPerm_Liv_F * C_CisPerm_Liv_F;

**# Rate of formation of metabolites - fetus**

dt(A_CisPerm_metabo_F) = r_CisPerm_metabo_Blood_F + r_CisPerm_metabo_Liv_F;

**# Rate of elimination (metabolism + transfer to placenta) - fetus**

dt(A_CisPerm_Elim_F) = dt(A_CisPerm_metabo_F) + dt(A_CisPerm_Trans_Pla);

**# Differential for amounts in tissues - fetus**

*# Liver*

dt(ACisPerm_Liv_F) = QLiv_F * (Ca_CisPerm_F - Cout_CisPerm_Liv_F)

- r_CisPerm_metabo_Liv_F;

*# Rest of the body*

dt(A_CisPerm_RB_F) = QRB_F *(Ca_CisPerm_F - Cout_CisPerm_RB_F);

*# Diffusion limited compartments*

dt(A_CisPerm_Int_Brain_F) = PS_Brain_F_CisPerm *(C_CisPerm_Ext_Brain_F

- Cout_CisPerm_Brain_F);

dt(A_CisPerm_Ext_Brain_F) = QBrain_F *(Ca_CisPerm_F - C_CisPerm_Ext_Brain_F)

+ PS_Brain_F_CisPerm *(Cout_CisPerm_Brain_F - C_CisPerm_Ext_Brain_F);

**# Venous and arterial blood**

Cv_CisPerm_F = (((QBrain_F * C_CisPerm_Ext_Brain_F) + (QLiv_F * Cout_CisPerm_Liv_F)

+ (QRB_F * Cout_CisPerm_RB_F))/QC_F);

dt(ACisPerm_Blood_F) = QC_F * (Cv_CisPerm_F -Ca_CisPerm_F) - r_CisPerm_to_placenta +

r_CisPerm_to_foetus - r_CisPerm_metabo_Blood_F;

**# Dynamics – *trans*-permethrin**

############################

**# Concentrations - dam**

*# Extracellular (venous) tissue space concentrations for diffusion limited compartments*

C_TransPerm_Ext_Kid = ATransPerm_Ext_Kid / V_Ext_Kid;

C_TransPerm_Ext_Fat = ATransPerm_Ext_Fat / V_Ext_Fat;

C_TransPerm_Ext_Mam = ATransPerm_Ext_Mam / V_Ext_Mam;

C_TransPerm_Ext_Brain = ATransPerm_Ext_Brain / V_Ext_Brain;

C_TransPerm_Ext_Sp = ATransPerm_Ext_Sp / V_Ext_Sp;

C_TransPerm_Ext_Musc = ATransPerm_Ext_Musc / V_Ext_Musc;

*# Intracellular concentrations for diffusion limited compartments*

C_TransPerm_Int_Kid = ATransPerm_Int_Kid / V_Int_Kid;

C_TransPerm_Int_Fat = ATransPerm_Int_Fat / V_Int_Fat;

C_TransPerm_Int_Mam = ATransPerm_Int_Mam / V_Int_Mam;

C_TransPerm_Int_Brain = ATransPerm_Int_Brain / V_Int_Brain;

C_TransPerm_Int_Sp = ATransPerm_Int_Sp / V_Int_Sp;

C_TransPerm_Int_Musc = ATransPerm_Int_Musc / V_Int_Musc;

*# Tissue concentrations for GI and Liver*

# C_TransPerm_GI = ATransPerm_GI / VGI;

# C_TransPerm_Liv = ATransPerm_Liv / VLiv;

*# Concentrations (Cout) at organ exit*

# flow-limited compartments

Cout_TransPerm_GI = ATransPerm_GI / (VGI * PC_GI_TransPerm);

Cout_TransPerm_Liv = ATransPerm_Liv / (VLiv * PC_Liv_TransPerm);

Cout_TransPerm_Rp = ATransPerm_Rp / (VRp * PC_Rp_TransPerm);

Cout_TransPerm_Pla = ATransPerm_Pla / (VPla * PC_Pla_TransPerm);

#diffusion limited compartments

Cout_TransPerm_Kid = C_TransPerm_Int_Kid / PC_Kid_TransPerm;

Cout_TransPerm_Fat = C_TransPerm_Int_Fat / PC_Fat_TransPerm;

Cout_TransPerm_Mam = C_TransPerm_Int_Mam / PC_Mam_TransPerm;

Cout_TransPerm_Brain = C_TransPerm_Int_Brain / PC_Brain_TransPerm;

Cout_TransPerm_Sp = C_TransPerm_Int_Sp / PC_Sp_TransPerm;

Cout_TransPerm_Musc = C_TransPerm_Int_Musc / PC_Musc_TransPerm;

*# Arterial blood concentration*

Ca_TransPerm = ATransPerm_Blood / VBlood;

**# Concentrations – fetus (whole litter)**

*# Extracellular (venous) tissue space concentrations for diffusion limited compartments*

C_TransPerm_Ext_Brain_F = A_TransPerm_Ext_Brain_F / V_Ext_Brain_F;

*# Intracellular (tissue) concentrations for diffusion limited compartments*

C_TransPerm_Int_Brain_F = A_TransPerm_Int_Brain_F / V_Int_Brain_F;

*# Tissue concentration for liver*

C_TransPerm_Liv_F = ATransPerm_Liv_F / VLiv_F;

*# Tissue concentration for rest of the body*

C_TransPerm_RB_F = A_TransPerm_RB_F / VRB_F;

*# Venous blood concentrations at organ exit*

# Diffusion limited compartments

Cout_TransPerm_Brain_F = C_TransPerm_Int_Brain_F / PC_Brain_F;

# Flow limited compartments

Cout_TransPerm_Liv_F = ATransPerm_Liv_F / (VLiv_F * PC_Liv_F);

Cout_TransPerm_RB_F = A_TransPerm_RB_F / PC_RB_F;

*# Arterial blood concentration*

Ca_TransPerm_F = ATransPerm_Blood_F / VBlood_F;

**# Rate of absorption**

dt(ATransPerm_gav) = DTransPerm_gav * BW;

dt(ATransPerm_GI1) = (DTransPerm_gav * BW)-(KsI_TransPerm * ATransPerm_GI1);

dt(ATransPerm_GI2) = (KsI_TransPerm * ATransPerm_GI1) - (KaI_TransPerm * ATransPerm_GI2)

- (KFec_TransPerm * ATransPerm_GI2);

dt(ATransPerm_abs) = KaI_TransPerm * ATransPerm_GI2;

**# Rate of metabolism - dam**

r_TransPerm_metabo_GI = CL_TransPerm_GI * Cout_TransPerm_GI;

r_TransPerm_metabo_Blood = CL_TransPerm_Blood * Ca_TransPerm;

r_TransPerm_metabo_Liv = CL_TransPerm_Liv * Cout_TransPerm_Liv;

**# Rate of formation of metabolites - dam**

dt(A_TransPerm_metabo) = r_TransPerm_metabo_GI + r_TransPerm_metabo_Blood

+ r_TransPerm_metabo_Liv;

**# Rate of Excretion**

dt(A_TransPerm_Fec) = KFec_TransPerm * ATransPerm_GI2;

dA_TransPerm_Fec_old = ((t_voiding!=0) ? A_TransPerm_Fec : dA_TransPerm_Fec_old);

t_old = (t_voiding!=0?t:t_old);

dA_TransPerm_Fec = ((t-t_old)>0 ? A_TransPerm_Fec-dA_TransPerm_Fec_old :

dA_TransPerm_Fec );

**# Rate of placenta to fetus transfer**

r_TransPerm_to_foetus = KTrans1 * Cout_TransPerm_Pla;

dt(A_TransPerm_Trans_Fet) = KTrans1 * Cout_TransPerm_Pla;

**# Rate of fetus to placenta transfer**

r_TransPerm_to_placenta = KTrans2 * Ca_TransPerm_F;

dt(A_TransPerm_Trans_Pla) = KTrans2 * Ca_TransPerm_F;

**# Rate of elimination - dam**

dt(A_TransPerm_Elim) = dt(A_TransPerm_metabo) + dt(A_TransPerm_Fec)

+ dt(A_TransPerm_Trans_Fet) - dt(A_TransPerm_Trans_Pla);

**# Differential for amounts in tissues - dam**

# Flow-limited compartments without metabolism

dt(ATransPerm_Rp) = QRp * (Ca_TransPerm - Cout_TransPerm_Rp);

# Diffusion limited compartments

*# Kidney*

dt(ATransPerm_Int_Kid) = PS_Kid * (C_TransPerm_Ext_Kid - Cout_TransPerm_Kid);

dt(ATransPerm_Ext_Kid) = QKid * (Ca_TransPerm - C_TransPerm_Ext_Kid)

+ PS_Kid * (Cout_TransPerm_Kid - C_TransPerm_Ext_Kid);

*# Muscles*

dt(ATransPerm_Int_Musc) = PS_Musc_TransPerm* (C_TransPerm_Ext_Musc -

Cout_TransPerm_Musc);

dt(ATransPerm_Ext_Musc) = QMusc * (Ca_TransPerm - C_TransPerm_Ext_Musc)

+ PS_Musc_TransPerm* (Cout_TransPerm_Musc - C_TransPerm_Ext_Musc);

*# Fat*

dt(ATransPerm_Int_Fat) = PS_Fat_TransPerm* (C_TransPerm_Ext_Fat - Cout_TransPerm_Fat);

dt(ATransPerm_Ext_Fat) = QFat * (Ca_TransPerm - C_TransPerm_Ext_Fat)

+ PS_Fat_TransPerm* (Cout_TransPerm_Fat - C_TransPerm_Ext_Fat);

*# Mam*

dt(ATransPerm_Int_Mam) = PS_Mam_TransPerm * (C_TransPerm_Ext_Mam -

Cout_TransPerm_Mam);

dt(ATransPerm_Ext_Mam) = QMam * (Ca_TransPerm - C_TransPerm_Ext_Mam)

+ PS_Mam_TransPerm * (Cout_TransPerm_Mam - C_TransPerm_Ext_Mam);

*# Brain*

dt(ATransPerm_Int_Brain) = PS_Brain_TransPerm * (C_TransPerm_Ext_Brain -

Cout_TransPerm_Brain);

dt(ATransPerm_Ext_Brain) = QBrain * (Ca_TransPerm - C_TransPerm_Ext_Brain)

+ PS_Brain_TransPerm * (Cout_TransPerm_Brain - C_TransPerm_Ext_Brain);

*# Sp*

dt(ATransPerm_Int_Sp) = PS_Sp_TransPerm *(C_TransPerm_Ext_Sp - Cout_TransPerm_Sp);

dt(ATransPerm_Ext_Sp) = QSp *(Ca_TransPerm - C_TransPerm_Ext_Sp)

+ PS_Sp_TransPerm *(Cout_TransPerm_Sp - C_TransPerm_Ext_Sp);

# Gastro-intestinal

dt(ATransPerm_GI) = QGI * (Ca_TransPerm - Cout_TransPerm_GI) + dt(ATransPerm_abs)

- r_TransPerm_metabo_GI;

# Liver

dt(ATransPerm_Liv) = (QLiv * Ca_TransPerm) + (QGI * Cout_TransPerm_GI)

- (QLiv_out * Cout_TransPerm_Liv) - r_TransPerm_metabo_Liv;

# Placenta

dt(ATransPerm_Pla) = QPla * (Ca_TransPerm - Cout_TransPerm_Pla) -r_TransPerm_to_foetus + r_TransPerm_to_placenta;

**# Venous and arterial blood - dam**

Cv_TransPerm = ((QLiv_out * Cout_TransPerm_Liv)

+ (QMusc * C_TransPerm_Ext_Musc) + (QKid * C_TransPerm_Ext_Kid)

+ (QFat * C_TransPerm_Ext_Fat) + (QMam * C_TransPerm_Ext_Mam)

+ (QBrain * C_TransPerm_Ext_Brain) + (QSp * C_TransPerm_Ext_Sp)

+ (QRp * Cout_TransPerm_Rp)

+ (QPla * Cout_TransPerm_Pla))/QC;

dt(ATransPerm_Blood) = QC * (Cv_TransPerm -Ca_TransPerm) - r_TransPerm_metabo_Blood;

**# Rate of metabolism – fetus**

r_TransPerm_metabo_Blood_F = CL_TransPerm_Blood_F * Ca_TransPerm_F;

r_TransPerm_metabo_Liv_F = CL_TransPerm_Liv_F * C_TransPerm_Liv_F;

**# Rate of formation of metabolites - fetus**

dt(A_TransPerm_metabo_F) = r_TransPerm_metabo_Blood_F + r_TransPerm_metabo_Liv_F;

**# Rate of Elimination (metabolism + transfer to placenta) - fetus**

dt(A_TransPerm_Elim_F) = dt(A_TransPerm_metabo_F) + dt(A_TransPerm_Trans_Pla);

**# Differential for amounts in tissues - fetus**

*# Liver*

dt(ATransPerm_Liv_F) = QLiv_F * (Ca_TransPerm_F - Cout_TransPerm_Liv_F)

- r_TransPerm_metabo_Liv_F;

*# Rest of the body*

dt(A_TransPerm_RB_F) = QRB_F *(Ca_TransPerm_F - Cout_TransPerm_RB_F);

*# Diffusion limited compartment*

dt(A_TransPerm_Int_Brain_F) = PS_Brain_F_TransPerm *(C_TransPerm_Ext_Brain_F –

Cout_TransPerm_Brain_F);

dt(A_TransPerm_Ext_Brain_F)= QBrain_F *(Ca_TransPerm_F - C_TransPerm_Ext_Brain_F)

+ PS_Brain_F_TransPerm *(Cout_TransPerm_Brain_F - C_TransPerm_Ext_Brain_F);

**# Venous and arterial blood - fetus**

Cv_TransPerm_F = ((QBrain_F * C_TransPerm_Ext_Brain_F) + (QLiv_F *

Cout_TransPerm_Liv_F) + (QRB_F * Cout_TransPerm_RB_F))/QC_F;

dt(ATransPerm_Blood_F) = QC_F * (Cv_TransPerm_F -Ca_TransPerm_F)

+ r_TransPerm_to_foetus - r_TransPerm_to_placenta

- r_TransPerm_metabo_Blood_F;

**# Check mass balance– *cis*-permethrin**

#################################

**# Dam**

ACisPerm_Tissue = ACisPerm_GI + ACisPerm_Liv

+ ACisPerm_Int_Fat + ACisPerm_Ext_Fat

+ ACisPerm_Int_Mam + ACisPerm_Ext_Mam

+ ACisPerm_Int_Brain + ACisPerm_Ext_Brain

+ ACisPerm_Int_Sp + ACisPerm_Ext_Sp

+ ACisPerm_Int_Musc + ACisPerm_Ext_Musc

+ ACisPerm_Rp

+ ACisPerm_Int_Kid + ACisPerm_Ext_Kid

+ ACisPerm_Pla

+ ACisPerm_Blood;

ACisPerm_Total = ACisPerm_Tissue + ACisPerm_GI1 + ACisPerm_GI2 + A_CisPerm_Elim ;

ACisPerm_Check = ACisPerm_gav - ACisPerm_Total;

**# Fetus**

ACisPerm_Tissue_F = A_CisPerm_Int_Brain_F + A_CisPerm_Ext_Brain_F

+ A_CisPerm_RB_F

+ ACisPerm_Blood_F + ACisPerm_Liv_F;

ACisPerm_Check_F = A_CisPerm_Trans_Fet - A_CisPerm_Elim_F - ACisPerm_Tissue_F;

**# Check mass balance– *trans*-permethrin**

###################################

**# Dam**

ATransPerm_Tissue = ATransPerm_GI + ATransPerm_Liv

+ ATransPerm_Int_Fat + ATransPerm_Ext_Fat

+ ATransPerm_Int_Mam + ATransPerm_Ext_Mam

+ ATransPerm_Int_Brain + ATransPerm_Ext_Brain

+ ATransPerm_Int_Sp + ATransPerm_Ext_Sp

+ ATransPerm_Int_Musc + ATransPerm_Ext_Musc

+ ATransPerm_Rp

+ ATransPerm_Int_Kid + ATransPerm_Ext_Kid

+ ATransPerm_Pla

+ ATransPerm_Blood;

ATransPerm_Total = ATransPerm_Tissue + ATransPerm_GI1 + ATransPerm_GI2

+ A_TransPerm_Elim ;

ATransPerm_Check = ATransPerm_gav - ATransPerm_Total;

**# Fetus**

ATransPerm_Tissue_F = A_TransPerm_Int_Brain_F + A_TransPerm_Ext_Brain_F

+ A_TransPerm_RB_F

+ ATransPerm_Blood_F + ATransPerm_Liv_F; ATransPerm_Check_F = A_TransPerm_Trans_Fet - A_TransPerm_Elim_F - ATransPerm_Tissue_F;

} # End of Dynamics

#################################################################################

**CalcOutputs {**

**# Blood and tissue concentrations - Dam (microgrammes/L)**

C_CisPerm_Blood = (ACisPerm_Blood ? ACisPerm_Blood / VBlood : 1E-10);

C_TransPerm_Blood = (ATransPerm_Blood ? ATransPerm_Blood / VBlood : 1E-10);

C_CisPerm_Liv = (ACisPerm_Liv ? ACisPerm_Liv / VLiv : 1E-10);

C_TransPerm_Liv = (ATransPerm_Liv ? ATransPerm_Liv / VLiv : 1E-10);

C_CisPerm_Pla = (ACisPerm_Pla ? ACisPerm_Pla / VPla : 1E-10);

C_TransPerm_Pla = (ATransPerm_Pla ? ATransPerm_Pla / VPla : 1E-10);

C_CisPerm_Rp = (ACisPerm_Rp ? ACisPerm_Rp / VRp : 1E-10);

C_TransPerm_Rp = (ATransPerm_Rp ? ATransPerm_Rp / VRp : 1E-10);

C_CisPerm_Kid = ( (ACisPerm_Int_Kid + ACisPerm_Ext_Kid) ?

(ACisPerm_Int_Kid + ACisPerm_Ext_Kid)/ VKid : 1E-10);

C_TransPerm_Kid = ( (ATransPerm_Int_Kid + ATransPerm_Ext_Kid) ?

(ATransPerm_Int_Kid + ATransPerm_Ext_Kid) / VKid : 1E-10);

C_CisPerm_Musc = ( (ACisPerm_Int_Musc + ACisPerm_Ext_Musc) ?

(ACisPerm_Int_Musc + ACisPerm_Ext_Musc)/ VMusc : 1E-10);

C_TransPerm_Musc = ( (ATransPerm_Int_Musc + ATransPerm_Ext_Musc) ?

(ATransPerm_Int_Musc + ATransPerm_Ext_Musc) / VMusc : 1E-10);

C_CisPerm_Fat = ( (ACisPerm_Int_Fat + ACisPerm_Ext_Fat) ?

(ACisPerm_Int_Fat + ACisPerm_Ext_Fat)/ VFat : 1E-10);

C_TransPerm_Fat = ( (ATransPerm_Int_Fat + ATransPerm_Ext_Fat) ?

(ATransPerm_Int_Fat + ATransPerm_Ext_Fat) / VFat : 1E-10);

C_CisPerm_Mam = ((ACisPerm_Int_Mam + ACisPerm_Ext_Mam) ?

(ACisPerm_Int_Mam + ACisPerm_Ext_Mam) / VMam : 1E-10);

C_TransPerm_Mam = ((ATransPerm_Int_Mam + ATransPerm_Ext_Mam) ?

(ATransPerm_Int_Mam + ATransPerm_Ext_Mam) / VMam : 1E-10);

C_CisPerm_Brain = ((ACisPerm_Int_Brain + ACisPerm_Ext_Brain) ?

(ACisPerm_Int_Brain + ACisPerm_Ext_Brain)/ VBrain : 1E-10);

C_TransPerm_Brain = ((ATransPerm_Int_Brain + ATransPerm_Ext_Brain) ?

(ATransPerm_Int_Brain + ATransPerm_Ext_Brain) / VBrain: 1E-10);

C_CisPerm_Sp = ((ACisPerm_Int_Sp + ACisPerm_Ext_Sp) ?

(ACisPerm_Int_Sp + ACisPerm_Ext_Sp) / VSp : 1E-10);

C_TransPerm_Sp = ((ATransPerm_Int_Sp + ATransPerm_Ext_Sp) ?

(ATransPerm_Int_Sp + ATransPerm_Ext_Sp) / VSp : 1E-10);

**# Blood and tissue concentrations - Fetus (microgrammes/L)**

C_CisPerm_Blood_F = (ACisPerm_Blood_F ? ACisPerm_Blood_F / VBlood_F : 1E-10);

C_TransPerm_Blood_F = (ATransPerm_Blood_F ? ATransPerm_Blood_F / VBlood_F : 1E-10);

C_CisPerm_Liv_F = (ACisPerm_Liv_F ? ACisPerm_Liv_F / VLiv_F : 1E-10);

C_TransPerm_Liv_F = (ATransPerm_Liv_F ? ATransPerm_Liv_F / VLiv_F : 1E-10);

C_CisPerm_RB_F = (A_CisPerm_RB_F ? A_CisPerm_RB_F / VRB_F : 1E-10);

C_TransPerm_RB_F = (A_TransPerm_RB_F ? A_TransPerm_RB_F / VRB_F : 1E-10);

C_CisPerm_Brain_F = ((A_CisPerm_Int_Brain_F + A_CisPerm_Ext_Brain_F) ?

(A_CisPerm_Int_Brain_F + A_CisPerm_Ext_Brain_F)/ VBrain_F : 1E-10);

C_TransPerm_Brain_F = ((A_TransPerm_Int_Brain_F + A_TransPerm_Ext_Brain_F) ?

(A_TransPerm_Int_Brain_F + A_TransPerm_Ext_Brain_F)/ VBrain_F : 1E-10);

}

End.
